# Anatomical and histological analyses reveal that tail repair is coupled with regrowth in wild-caught, juvenile American alligators (*Alligator mississippiensis*)

**DOI:** 10.1038/s41598-020-77052-8

**Published:** 2020-11-18

**Authors:** Cindy Xu, Joanna Palade, Rebecca E. Fisher, Cameron I. Smith, Andrew R. Clark, Samuel Sampson, Russell Bourgeois, Alan Rawls, Ruth M. Elsey, Jeanne Wilson-Rawls, Kenro Kusumi

**Affiliations:** 1 School of Life Sciences, Arizona State University, P.O. Box 874501, Tempe, AZ 85287 USA; 2 Department of Basic Medical Sciences, University of Arizona College of Medicine-Phoenix, Phoenix, AZ 85004 USA; 3 Russell Bourgeois, Jeanerette, LA 70544 USA; 4 Rockefeller Wildlife Refuge, Louisiana Department of Wildlife and Fisheries, Grand Chenier, LA 70643 USA

**Keywords:** Regeneration, Non-model organisms, Zoology

## Abstract

Reptiles are the only amniotes that maintain the capacity to regenerate appendages. This study presents the first anatomical and histological evidence of tail repair with regrowth in an archosaur, the American alligator. The regrown alligator tails constituted approximately 6–18% of the total body length and were morphologically distinct from original tail segments. Gross dissection, radiographs, and magnetic resonance imaging revealed that caudal vertebrae were replaced by a ventrally-positioned, unsegmented endoskeleton. This contrasts with lepidosaurs, where the regenerated tail is radially organized around a central endoskeleton. Furthermore, the regrown alligator tail lacked skeletal muscle and instead consisted of fibrous connective tissue composed of type I and type III collagen fibers. The overproduction of connective tissue shares features with mammalian wound healing or fibrosis. The lack of skeletal muscle contrasts with lizards, but shares similarities with regenerated tails in the tuatara and regenerated limbs in *Xenopus* adult frogs, which have a cartilaginous endoskeleton surrounded by connective tissue, but lack skeletal muscle. Overall, this study of wild-caught, juvenile American alligator tails identifies a distinct pattern of wound repair in mammals while exhibiting features in common with regeneration in lepidosaurs and amphibia.

## Introduction

Appendage regeneration is widespread among vertebrate groups and anamniotes, such as the zebrafish, *Xenopus*, and axolotl, have long been the focus of regenerative studies. Among amniotes, non-avian reptiles are the only group known to regenerate complex, multi-tissue structures such as the tail^1–4^, whereas mammals and birds exhibit a very limited capacity for regeneration as adults. In contrast to regeneration-enabled vertebrates, the mammalian injury response is characterized by slow, wound healing and repair of damaged tissues^5^. Compared to normal tissue, scar tissue has reduced functionality, decreased sensitivity, and greater risk of infection^6^. As a result, many researchers are dedicated to understanding the mechanisms, as well as evolutionary pressures, that enable structural regeneration in different vertebrate taxa, which can be utilized to enhance or improve wound healing outcomes in mammals.

Selective pressures are driving forces of trait evolution, and within some, but not all taxa, predation pressures are likely associated with the maintenance of regenerative abilities. Among vertebrates, sublethal predation is common among natural populations of teleost fish, anuran tadpoles, urodele amphibians, and non-avian reptiles^7–12^. As a result, some species of salamanders and lizards evolved the ability to autotomize, or self-amputate, the tail as an evasive defense tactic^13–21^. Moreover, skin autotomy has also been reported in a rodent, the African spiny mouse^22^. While an obvious advantage of autotomy is immediate survival, the absence of a fin, limb, or tail can have severe repercussions for an individual, as these structures are essential for locomotion and balance, energy storage, sexual selection, and defense^23–29^. However, the regeneration of lost structures can mitigate these costs by recovering partial or full function of the original structure^21,28,30,31^. While regeneration is selectively advantageous, closely-related taxa can demonstrate a wide range of regenerative abilities, including some with limited or no capacity. In these instances, regenerative capacity may be lost as a neutral trait or by negative selection as a trade-off of energy allocation, reproduction, or developmental growth^32^. In mammals, it is hypothesized that the loss of regenerative capacity may be related to the development of a specialized immune system^33,34^, increased regulation of the cell cycle^35^, or the evolution of endothermy^36^.

**Figure 1:**
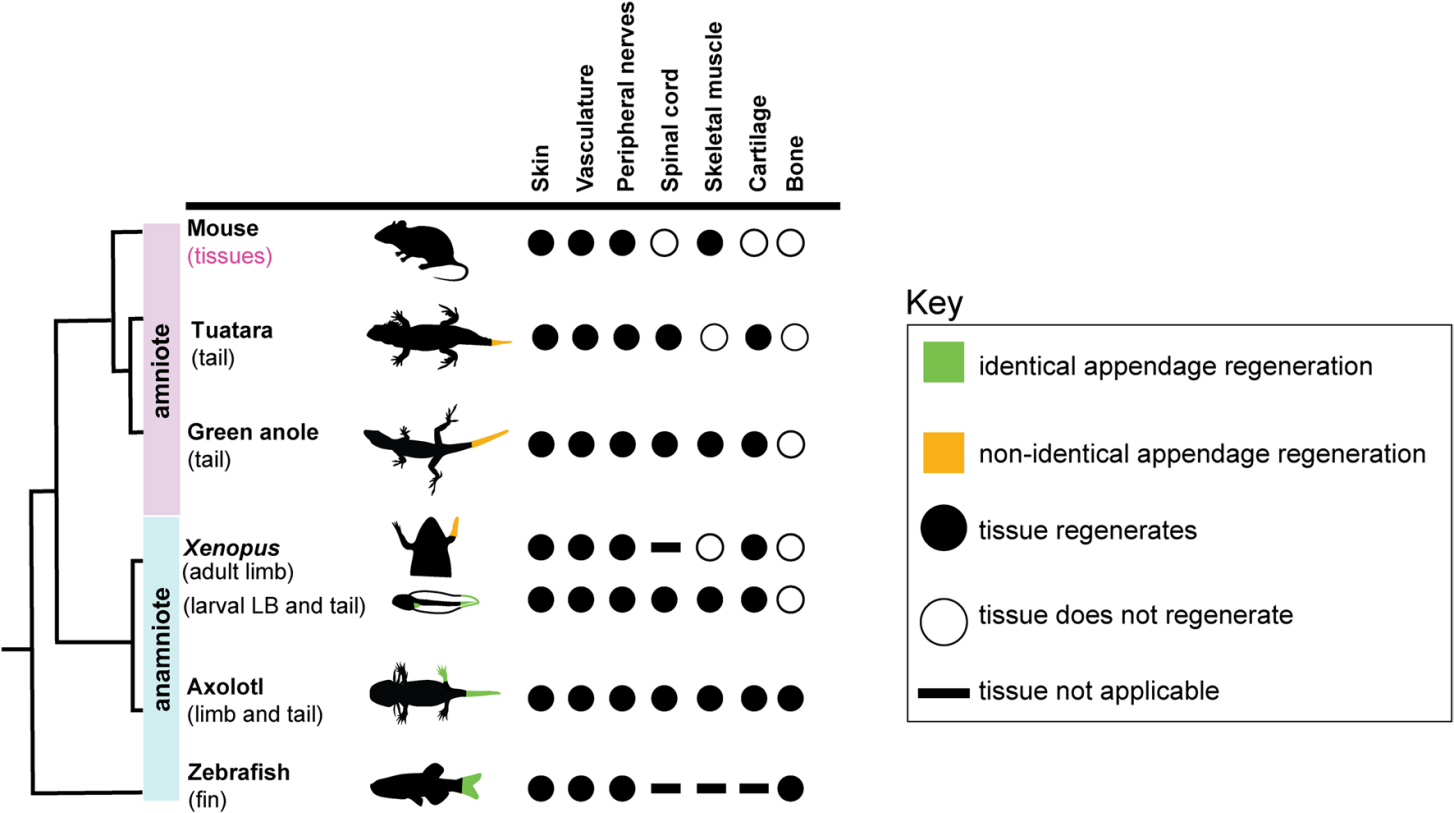
Vertebrate appendage regeneration or regrowth is widespread but variable. Schematic summarizing appendage regeneration or regrowth in vertebrates. For mouse, regeneration is only observed in limited tissues, but not in appendages. LB-limb bud.

Processes such as a diminished injury response, specialized wound epidermis formation, extracellular matrix remodeling, reinnervation, and reactivation of conserved developmental pathways are shared across regeneration-enabled vertebrates^37–39^, indicating a conserved program that is likely altered or shut down in mammals. On the other hand, regenerative capacity of vertebrate appendages varies extensively and can be viewed as a spectrum of abilities. Whereas zebrafish, axolotls, and larval *Xenopus* frogs are capable of regrowing structures nearly identical to the original appendage and adult *Xenopus* frogs and lepidosaurs exhibit non-identical regeneration, mammals do not replace lost appendages (Fig. 1). Rather, mammals can regenerate tissues such as skeletal muscle or peripheral nerves, but the majority of mammalian tissues or organs cannot be replaced (Fig. 1). Given the variation in size, shape, and tissues (Fig. 1), different taxa have likely evolved divergent strategies of regeneration and warrant further study. For example, Prod1 is involved in cell adhesion and positional identity during limb regeneration, but is specific only to salamanders^40–42^. Also lizards, but not the tuatara, are able to regenerate skeletal muscle de novo^3,43,44^. Regenerative capacity is also contingent upon an animal’s life history traits including body size, growth patterns, life stage, and age^45^. While studies have established that increased body size can delay healing in newt limbs^46^ and different developmental life stages impact regenerative capacity of *Xenopus* limbs^47,48^*,* the influence of these traits remain understudied. Moreover, the vertebrate regenerative process has been extensively studied in model systems such as the zebrafish, *Xenopus*, axolotl, and lizards such as the green anole, but studies of regeneration in new animal systems are increasingly being published^49–55^. This provides further insight into the diversity of regenerative abilities within and between taxa and opens up opportunities for future comparative studies.

Reptiles exhibit a high degree of species richness, are distributed across a wide range of environmental niches, and have evolved a diverse set of life history, physiological, behavioral, and morphological traits^56–58^. Given that the tail is functionally necessary for locomotion and stability, defense, energy storage, and sexual selection^19^, environmental changes would greatly affect its structure and hence, regenerative abilities. Reptilia is represented by three extant infra-classes, Neodiapsida, which includes turtles, Archosauromorpha, which includes crocodilians and birds, and Lepidosauromorpha, or lepidosaurs, which includes squamates (lizards, snakes) and the tuatara^59,60^. Lepidosaurs, such as lizards, have evolved the ability to autotomize, or self-amputate, their tails and can regenerate a biomechanically functional tail^16,18,61^. Moreover, in lizard species with regenerative abilities, the regrown tail is anatomically distinct from the original structure formed during development^1,3,4,62–64^, distinguishing lizards from traditional anamniote models of regeneration. The core endoskeleton of the lizard regenerated tail consists of an unsegmented cartilage tube that encloses the ependyma and central descending axons^1–4^. No new neurons are produced during lizard tail regeneration, instead regrowing axons are derived from neurons located in the spinal cord and dorsal root ganglia that originate in the tail stump^65–69^. Additionally, regenerated skeletal muscles form longitudinal fibers that are radially arranged around the cartilage tube and lack organization^3^. However, tail regenerative capacity is variable within lepidosaurs. While snakes are not known to replace the tail after injury, the tuatara, like lizards, regrows a cartilaginous endoskeleton but exhibits minimal or no skeletal muscle. Rather, the majority of the regrown tuatara tail is composed of dense, connective tissue reminiscent of fibrotic tissue^44^.

Tail regeneration has been extensively studied in lepidosaurs, but there have been published reports that modern crocodilians (alligators, caimans, crocodiles, and gharials) are also capable of tail regrowth (Table 1). The regrowth process is considered to be slow, occurring over the span of many months^70,71^. Reports of crocodilian tail regrowth also describe the outward appearance of the regrown tail as different from the original tail^70–76^, and a study in the black caiman showed that the regrown tail segment does not reform caudal vertebrae^71^. However, to date there are no detailed descriptions of the tissues in the regrown crocodilian tail. Here, we provide the first anatomical and histological analysis of tails with abnormal morphology from wild-caught, juvenile alligators. We predict these tails were lost by traumatic injury and refer to the tails as reparative regrowth, or regrown tails for short. Given that non-avian reptiles are (1) a highly diverse group with different regenerative capacities and (2) the only amniotes with regenerative abilities, the characterization of structural repair and regrowth in non-avian reptiles, such as the American alligator, will serve as a valuable comparison point within amniotes to understand the mechanisms and fundamental traits that enable or limit reparative regrowth.

**Table 1:** Reports of tail regrowth in the order crocodilia.

Family	Species	Regenerated tail length (cm)	Reference
Alligatoridae	*Alligator mississippiensis*	–	Han et al.^74^
	*Caiman crocodilus* (Nr. 102/1911)	2.0	Kälin^72^
	*Caiman crocodilus* (Nr. 95/1911)	4.0	Kälin^72^
	*Caiman crocodilus* (Nr. 103/1911)	–	Kälin^72^
	*Caiman crocodilus*	7.0	Dathe^71^
	*Caiman crocodilus*	6.0	Voigt^70^
	*Caiman crocodilus*	9.0	Ramírez-Bravo et al.^76^
	*Melanosuchus niger *(Nr. 63/1911)	21.5	Kälin^72^
	*Melanosuchus niger*	30.0	Kälin^72^
	*Paleosuchus trigonatus*	4.0	Lemaire and Marquis^75^
Crocodilidae	*Crocodylus johnstoni*	–	Webb and Manolis^73^
Gavialidae	No reports of tail regrowth		

## Materials and methods

### Specimen collection and anatomical data acquisition

This study was approved by the Louisiana Department of Wildlife and Fisheries (LDWF) and is in accordance with the Louisiana Alligator Regulations (Louisiana Revised Statutes, Title 56, Part V. Wild Quadrupeds and Wild Birds, Chapter 7. Alligators, Section 701. Alligator Regulations) and the official regulations of the Louisiana Wildlife and Fisheries Commission and Best Management Practices guidelines. The approved procedures include the capture and humane sacrifice of wild American alligators as well as the collection and transport of alligator samples salvaged from individuals procured for other research studies at the Rockefeller Wildlife Refuge (Grand Chenier, LA) or identified as nuisance alligators removed from private property off site. All animals were handled by research biologists at the Rockefeller Wildlife Refuge or a nuisance alligator trapper licensed by the Louisiana Department of Wildlife and Fisheries (license number LA-26861). These procedures followed all ethical and legal recommendations proposed by the Louisiana Department of Wildlife and Fisheries.

Three regrown alligator tail segments (A01–A03) and an original specimen (A00), which was examined to confirm published reports on normal anatomy, were collected post-mortem and provided by LDWF research staff (Table 2, Supplementary Data S1). Regrown tail samples and the original specimen were salvaged within hours after death and preserved in 70% ethanol at 4 °C. Post-mortem alligator samples were transported to Arizona State University (Tempe, AZ) for further analysis and authorized by the Louisiana Department of Wildlife and Fisheries (special alligator permit—education/research use to KK, CX). The original specimen was fresh frozen for preservation and regrown tail segments were transferred to fresh 70% ethanol and rehydrated through a series of 24 h graded ethanol washes. Next, regrown tails were immersion fixed in 4% paraformaldehyde (PFA) at 4 °C with constant agitation and returned to 70% ethanol for long-term storage. Additional photographs as well as a radiograph and biopsy report of a fourth individual with a regrown tail (A04) were also analyzed (Supplementary Data S1–S3). All individuals sampled were juveniles or sub-adults. The condition under which the tails were lost and the duration of regrowth are unknown.

**Table 2:** Sample specifics for American alligator specimens analyzed with regrown tail segments.

Animal	Sex	Tail	Life Stage	Body condition score	Regrown tail segment length (cm)	Total body length (cm)	Proportion regrown tail segment/total body length (%)
A01	F	Regrown	Juvenile/sub-adult	3	24.1	133.4	18.1
A02	M	Regrown	Juvenile/sub-adult	3	11.4	179.1	6.5
A03	F	Regrown	Juvenile	3	11.4	104.1	11.0
A04	M	Regrown^a^	Juvenile	3	NR	63.5	NR

^a^Reparative outgrowth observed from dorsal surface and distal tip of tail. Numerical body condition scores (BCS) were assigned based on the physical condition of the limbs, spinal column, jowls, and tail girth^79^. Body condition scores are categorized as normal for their range (3), thin or slender (2), or emaciated (1). NR-not reported.

Radiographs were obtained for all tails with a MinXray HF8015 + DLP portable unit (Northbrook, IL) at 65 kVP at 5 mAs at Arizona State University (Tempe, AZ) and at Louisiana State University School of Veterinary Medicine (A04, Baton Rouge, LA). Magnetic resonance images (MRI) were acquired using a Bruker Biospec 7-T at the Barrow Neurological Institute Center for Preclinical Imaging (Phoenix, AZ) and at the University of Arizona College of Medicine (Tucson, AZ). MR images were collected for two regenerated tails (A01, A03) using a T1 weighted fast low-angle shot pulse sequence with the following parameters: TR/TE of 130/5.5–9 ms, flip angle of 45°, voxel size of 200 microns, and NEX of 6. Specimen A01 data were acquired with a FOV of 180 × 80 × 60 mm and matrix of 900 × 400 × 300. Specimen A03 data were acquired in 3 stations with a FOV of 83.2 × 83.2 × 51.2 mm and a matrix of 416 × 416 × 256. All MRI data were analyzed using Amira v6.4.0 (Visage Imaging, Berlin, Germany) and tissues were manually segmented to obtain volume measurements. Anatomical data for each specimen were obtained by gross dissection and muscles were numerically labeled. All stages of the dissection were photo-documented using a Canon EOS Rebel T3i camera.

### Terminology

Caudal tail muscles described below are referred to by their assigned muscle groups as there is currently no standardized nomenclature system that describes reptilian axial musculature. This is largely due to the lack of clarification regarding individual muscle subdivisions and their homologies, which have previously been summarized in the literature^77,78^. Muscle groups will be referred to as *M. transversospinalis, M. longissimus*, *M. ilio-ischiocaudalis*, and *M. caudofemoralis*, which will provide a general but consistent representation of the tail musculature in the American alligator.

### Tissue processing and paraffin embedding

Tissue samples were fixed overnight in 4% PFA at 4 °C with constant agitation before dehydration through a series of graded ethanol washes (PBS, 25%, 50%, 70%, 95%, 100%), cleared in xylenes, and embedded in paraffin. Samples were serially sectioned using a Leica RM2235 microtome, at 0.5 μm thickness, and mounted on charged Histobond slides (VWR, Radnor, PA). Slides were then cleared in xylenes to remove excess wax, briefly rehydrated through graded ethanol washes (100%, 80%, 70%, 50% and 25%), and rinsed in distilled H_2_O before staining. Methods for each staining procedure can be found below. Following histological staining, all slides were rinsed in diH_2_O, dehydrated, cleared in xylenes, and affixed with Permount (Fisher Scientific, SP15-100, Hampton, NH). Images were captured using an Olympus BX50 microscope.

### Histological techniques

#### Hematoxylin and eosin stain

Slides were stained in Harris modified hematoxylin (Ricca Chemical Company, 3530, Arlington, TX), rinsed in double deionized water (diH_2_O) and incubated in acid alcohol (0.4% HCl in 70% EtOH), followed by 1% ammonia diH_2_O. Slides were then partially dehydrated (25%, 50%), and counterstained with eosin. Hematoxylin stains nuclei purple, while eosin stains cytoplasm and extracellular matrix proteins pink. Highly hydrophobic structures and cells, like adipocytes, do not stain.

#### Gomori’s trichrome stain

Dehydrated slides were fixed in Bouin’s solution at 58 °C, rinsed thoroughly in tap H_2_O, then diH_2_O, incubated in Weigert’s hematoxylin, and rinsed in diH_2_O again. After a brief acid alcohol incubation, slides were stained in trichrome solution (acetic acid, phosphotungstic acid, chromotrope 2R, and fast green FCF), and incubated in 0.5% acetic acid diH_2_O. Collagen rich structures, such as basement membranes and fibrotic tissues stain blue-green, while cytoplasm stains red-purple and nuclei stain blue-black.

#### Picrosirius red stain

Dehydrated slides were stained in Weigert’s hematoxylin, followed by washing under running water. The slides were then incubated in 0.1% Sirius red picric acid solution for 1 h and rinsed in 0.5% acetic acid diH_2_O. Collagen stains red, while cytoplasm is pale yellow. If visible, the nuclei stain grey-black.

#### Herovici’s polychrome stain

Dehydrated slides were incubated in polychrome solution (methyl blue, acid fuchsin, acetic acid, picric acid), followed by 1% acetic acid diH_2_O. This preparation stains mature collagen type I fibers red-purple, and young collagen type III fibers blue. The cytoplasm is counterstained yellow, and the nuclei are blue-black.

### Immunohistochemistry

Monoclonal anti-myosin heavy chain antibody MY-32 (Sigma-Aldrich, M4276, St. Louis, MO), monoclonal anti-COL2A1 antibody II-II6B3 (DSHB, Iowa City, IA) and biotinylated goat broad spectrum IgG secondary antibody (Invitrogen, 859043, Carlsbad, CA) were used for immunohistochemistry (IHC), diluted according to the manufacturer’s suggestions. Slides were de-paraffinized in xylenes, rehydrated, and heated to 95 °C for 10 min in pH 6.0 citrate buffer for epitope retrieval. After cooling to room temperature, slides were permeabilized in 0.5% Triton X-100 PBS (PBS-T) and incubated 1 h in 10% goat serum (Invitrogen, 859043) to block non-specific binding. Slides were incubated overnight at 4 °C with primary antibody diluted in 1% serum PBS-T, washed with PBS-T, and subsequently incubated for 1 h at 37 °C with secondary antibody in 1% serum PBS-T. HRP Streptavidin solution was added for 30 min, followed by PBS-T washes, DAB substrate incubation, and hematoxylin counterstain. Antigen retrieval was not performed on cartilage sections, as the tissue detaches from the slide post citrate treatment. Images were captured using an Olympus BX50 microscope.

## Results

### External morphology and osteology of the original tail

**Figure 2:**
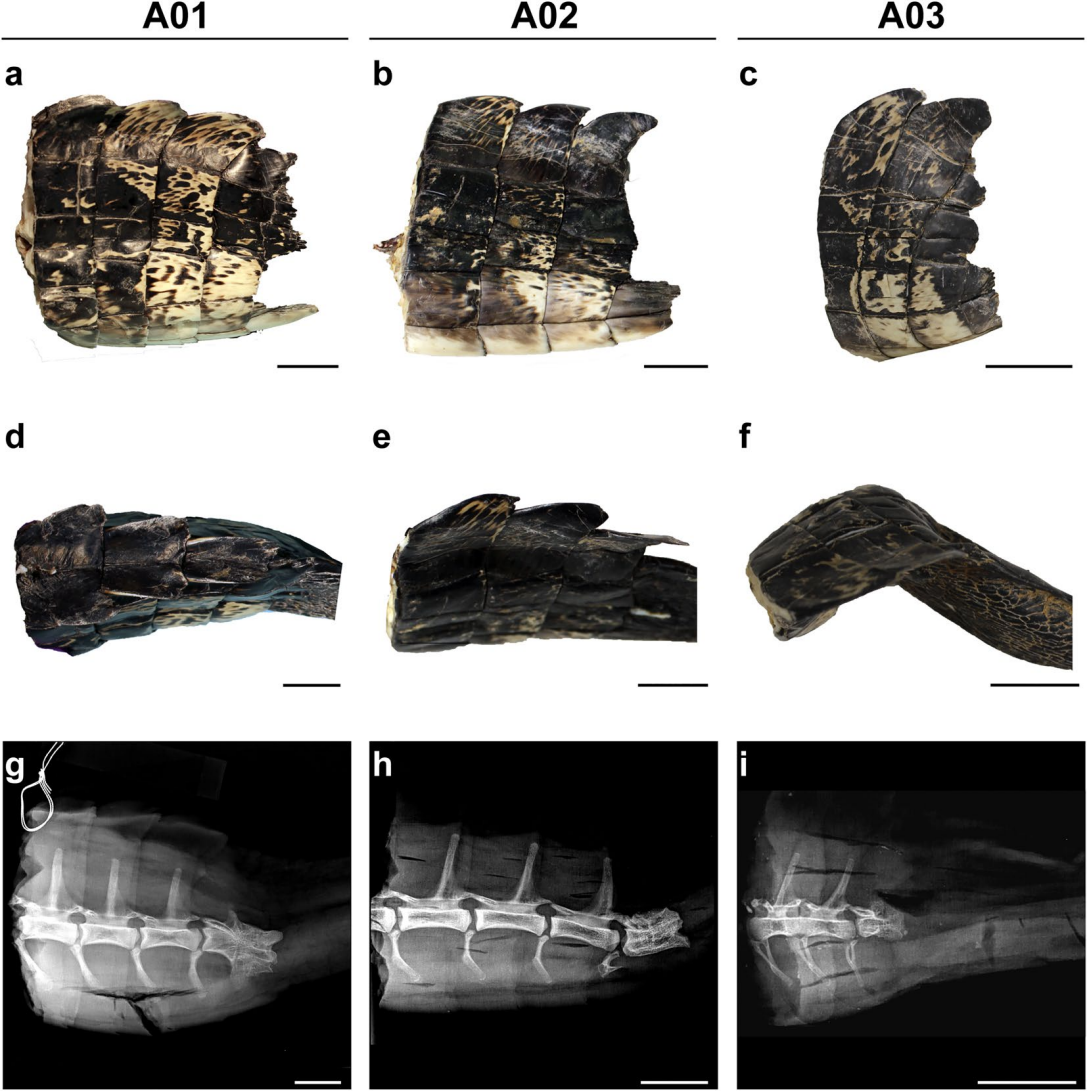
The original tail is covered in organized scales and caudal vertebrae exhibit prominent structures. Lateral views of original tail segments proximal to the site of injury (**a**–**c**). Scales are mottled and organized into transverse rows identified by dorsal scutes. Dorsal views of original tails (**d**–**f**). A01 exhibits paired dorsal scutes (**d**) while A02 and A03 exhibit single dorsal scutes (**e**,**f**). Lateral radiographs reveal articulated caudal vertebrae that feature prominent neural spines and hypapophyses (**g**–**i**). The last remaining caudal vertebra exhibits bone fissures and lacks vertebral processes. Scale bars are 2.5 cm.

Using anatomical and histological data, we carried out a comparative analysis of the original and regrown tail segment tissues located near the junction site in the American alligator. All samples analyzed were obtained from wild-caught, juvenile or sub-adult alligators of both sexes (2F:1M) and were assigned a body condition score of 3, as all individuals were well-nourished and in good physical condition at the time of capture^79^. Additional information, including size measurements, are presented in Table 2. Original tail segments were covered by non-overlapping, rectangular scales and dorsal scutes organized into transverse rows (Fig. 2a–c). The dorsal scales were mottled and darker in color when compared to the ventral scales (Fig. 2a–f). Among the samples analyzed, only specimen A01 exhibited paired dorsal scutes, indicating that A01 sustained a more proximal injury (Fig. 2d). Radiographs revealed that each proximal caudal vertebra corresponded with a single row of scales and featured elongated neural spines and hypophyses (Fig. 2g–i). The caudal vertebra located immediately proximal to the presumed injury site lacked these spinal processes and had bone fissures, indicating there was remodeling (Fig. 2g–i).

For comparative purposes, we analyzed an intact, original tail from a female, juvenile American alligator (Supplementary Figure S1). The axial skeleton of American alligators consists of 65 total vertebrae of which, 38–41 are caudal (Ca) vertebrae^80^. In the specimen analyzed (A00), we identified a total of 40 caudal vertebrae. Ca vertebrae 1–14 exhibited transverse processes, which is consistent with previous anatomical studies^81^. The vertebral column extended along the entire length of the tail and spinal processes gradually diminished towards the distal tip. Additionally, each caudal vertebrae corresponded to a single scale segment with paired dorsal scutes terminating at segment 18 (Supplementary Figure S1). Because only A01 exhibited paired dorsal scutes in the original tail segment (Fig. 2d), we estimated that this individual lost approximately half of the posterior tail. Samples, A02 and A03, exhibited only single dorsal scutes in the original tail segment, indicating that the tail was truncated distal to Ca 18 (Fig. 2e,f). By counting the scale rows starting at the base of the original tail segment, and by counting scale rows, we estimated that A02 and A03 tails were truncated near Ca 24 and 20, respectively. Crocodilian vertebrae do not possess autotomy planes^82^, nor were any observed in the alligator; thus, the substantial loss of the posterior tail was likely the result of traumatic amputation although birth defects cannot be ruled out.

### Anatomy and histology of skeletal muscles in the original tail

**Figure 3:**
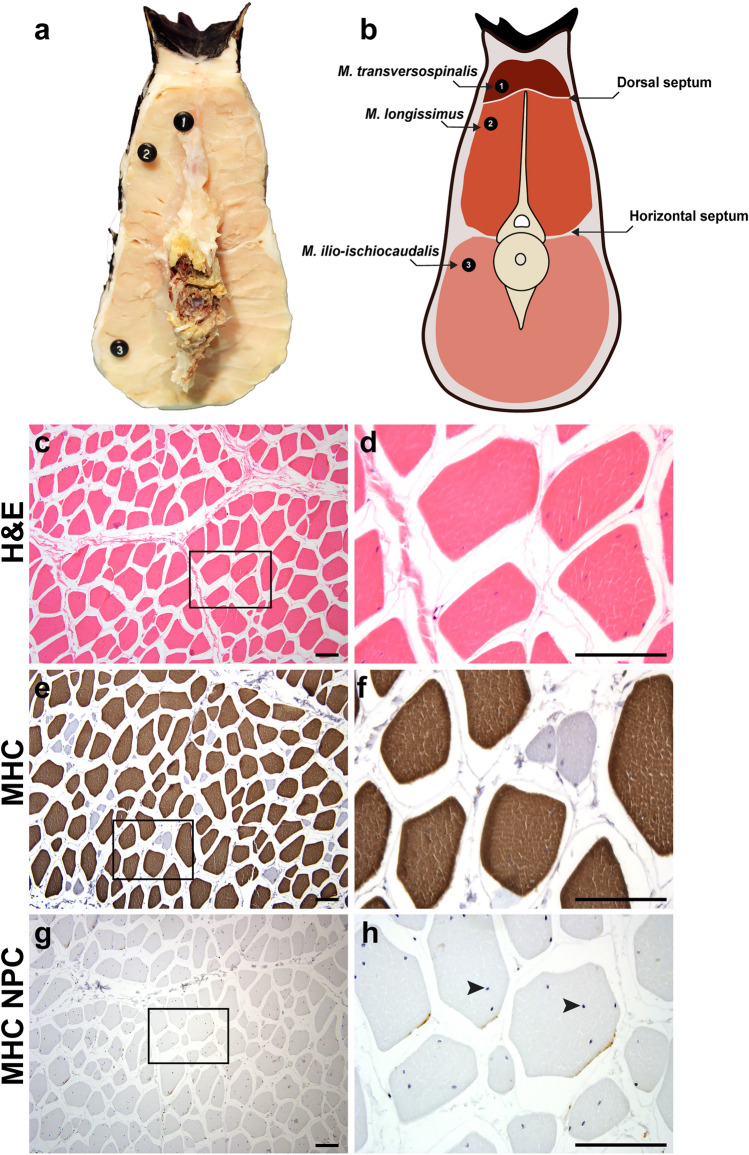
Original tail musculature is organized into distinct epaxial and hypaxial domains. Transverse view (**a**) and schematic diagram (**b**) of the caudal tail musculature. Epaxial muscles include *M. transversospinalis* (1) and *M. longissimus* (2), divided by a thin dorsal septum. The hypaxial muscle is composed of *M. ilio-ischiocaudalis* (3), which is separated from the epaxial domain by a thick horizontal septum. Sections were stained with H&E (**c**,**d**) or subjected to IHC with an anti-fast MHC antibody and counterstained with hematoxylin (**e**,**f**). Proximal to the site of injury, multinucleated myofibers are present (**c**,**d**), and as expected, the myofibers stain positive for MHC (**e**,**f**). Myonuclei can be visualized in MHC negative primary controls (**g**,**h**). Black arrowheads mark the myonuclei (h). Images are representative and scale bars are 100 µm. *MHC* myosin heavy chain, *NPC* no primary control.

Dissections of the proximal original tail revealed a large volume of muscle surrounding the vertebral column, which was bisected into distinct epaxial and hypaxial domains by a thick horizontal septum (Fig. 3a,b). The epaxial muscles consisted of *M. longissimus* and *M. transversospinalis*, which were separated by an intermuscular dorsal septum, also known as the *septum intermusculare dorsi*^83^. Whereas *M. longissimus* occupied a large portion of the epaxial domain, *M. transversospinalis* was relatively slender. Both muscles have been described as extending along the length of the tail, but tapering near the distal end, as well as interweaving of these muscles, make it difficult to distinguish between the two groups^84^. Indeed, we were unable to identify a distinct *M. transversospinalis* in the original tail in 2 of the 3 specimens dissected. The hypaxial muscle domain was solely composed of *M. ilio-ischiocaudalis* (Fig. 3a,b), which typically encloses *M. caudofemoralis* in the proximal tail region. However, the absence of *M. caudofemoralis* was expected in the three specimens analyzed, as amputation had occurred distal to the location of the transverse processes and *M. caudofemoralis*^85,86^. It is hypothesized that the transversospinalis muscle group functions as a stabilizer of the vertebral column, while unilateral or bilateral contraction of the epaxial longissimus and hypaxial ilio-ischiocaudalis muscle groups facilitate lateral or ventral flexion of the reptile tail^62,80^. Hematoxylin and eosin (H&E) staining of transverse sections from proximal muscle revealed uniform bundles of muscle fibers, surrounded by basement membrane that were organized in fascicles (Fig. 3c,d). IHC conducted using a broad species reactive antibody against fast myosin heavy chain (MHC), demonstrated that the muscle contained predominantly fast type fibers (Fig. 3e–h).

### External morphology of the regrown tail is distinct from the original

**Figure 4:**
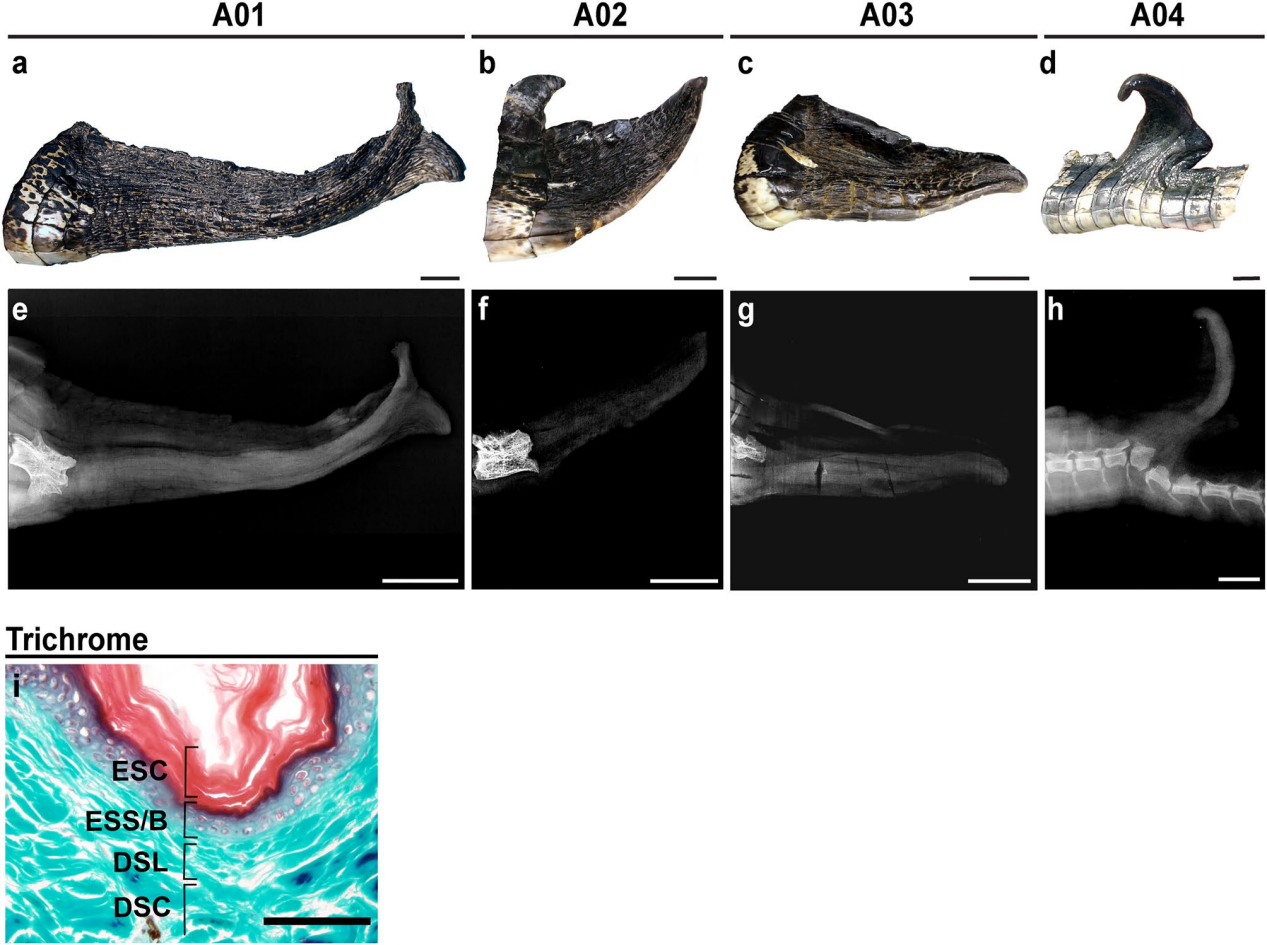
The regrown tail exhibits abnormal scale patterning and lacks caudal vertebrae. Photographs of regrown tail segments after full (**a**–**c**) or partial amputation (**d**) of the posterior tail. The regrown tail lacks dorsal scutes and re-patterned scales are small, black and uniformly distributed. Lateral radiographs demonstrate that caudal vertebrae do not regenerate but are replaced by a rod-like structure (**e**–**h**). Superficial samples of the regrown skin stained with Gomori’s Trichrome show all layers of the epidermis and the dermis are present (**i**). Images are representative and scale bars are 2.5 cm (**a**–**h**) or 100 µm (**i**). *ESC* epidermal stratum corneum, *ESS/B* epidermal stratum spinosum/basale, *DSL* dermal stratum laxum, *DSC* dermal stratum compactum.

Crocodilians can regenerate their tail but not their limbs, which is a similar pattern observed in lizards (Table 1, Supplementary Data S1, Supplementary Data S2). The average length of the regrown tails measured 15.7 ± 7.3 cm, which constituted approximately 6–18% of the totally body length (n = 3, Table 2), and regrown tail segments were easily identified by external morphology. Scales of the regrown tail differ in color and patterning relative to the original tail. Small, black scales were uniformly distributed around the circumference of the regenerated tail, which lacked dorsal scutes (Fig. 4a–d). These scales were strongly adhered to the underlying tissue. Transverse sections through the skin of the regrown tail showed all the typical layers of the epidermis and dermis were present (Fig. 4i), starting with the exterior epidermal stratum corneum, staining red with Gomori’s trichrome preparation, followed by the epidermis stratum spinosum and stratum basale, which gives rise to new keratinocytes. Finally, the underlying dermis was comprised of both loose and compacted layers (Fig. 4i).

### The regrown tail is supported by a cartilaginous endoskeleton

**Figure 5:**
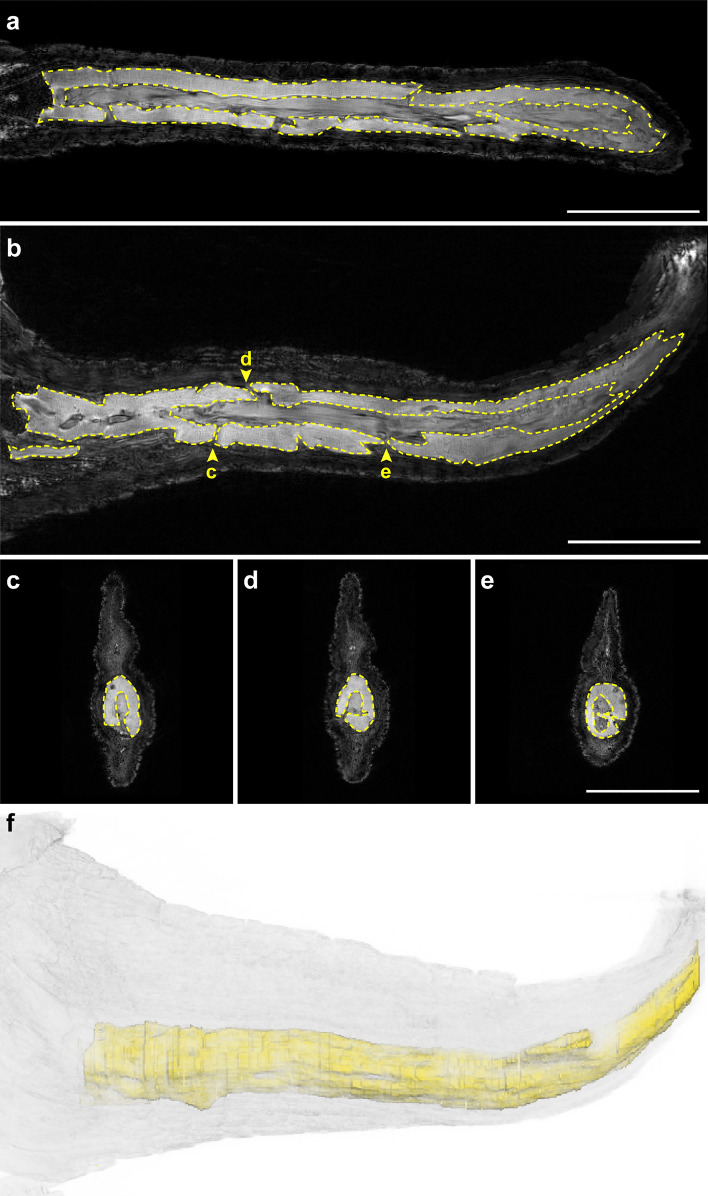
The endoskeleton of the regrown tail forms a hollow, unsegmented rod with randomly distributed foramina. Representative orthogonal magnetic resonance images (**a**–**e**) and 3D reconstruction (**f**) of a regrown alligator tail. The regrown endoskeleton forms a hollow tube that lacks segmentation (**a**,**b**). Foramina (**b**–**e**) are randomly distributed along the proximo-distal axis of the tail. The regrown endoskeleton is ventrally positioned in the tail (**c**–**f**). Scale bars are 2.5 cm.

Radiographs revealed that there was no bone in the regrown tail segment, but did detect the presence of a rod-like structure (Fig. 4e–g). In one example, this structure was observed in a regrown tail that protruded from the dorsal surface of the original tail (Fig. 4h). Such protrusions may occur following injuries that do not result in complete amputation of the original structure and is known to occur in other non-avian reptiles such as lizards^87–90^. This individual, A04, was also missing the distal tip of the tail and had regrown a small segment. Given that soft tissues exhibit subtle differences in density that cannot be differentiated by radiographs, we utilized magnetic resonance imaging (MRI) to further examine the morphology of this structure. MRI confirmed the presence of an unsegmented, hollowed, rod-like structure (Fig. 5a) with foramina distributed along the length of the tail (Fig. 5b–e, Supplementary Video S1). We found that similar foramina in the regenerated tail of the green anole lizard served as channels for regrowing blood vessels and axons^3,91^. The rod-like structure was ventrally positioned in the regrown alligator tail (Fig. 5c–f, Supplementary Video S2, S3).

**Figure 6:**
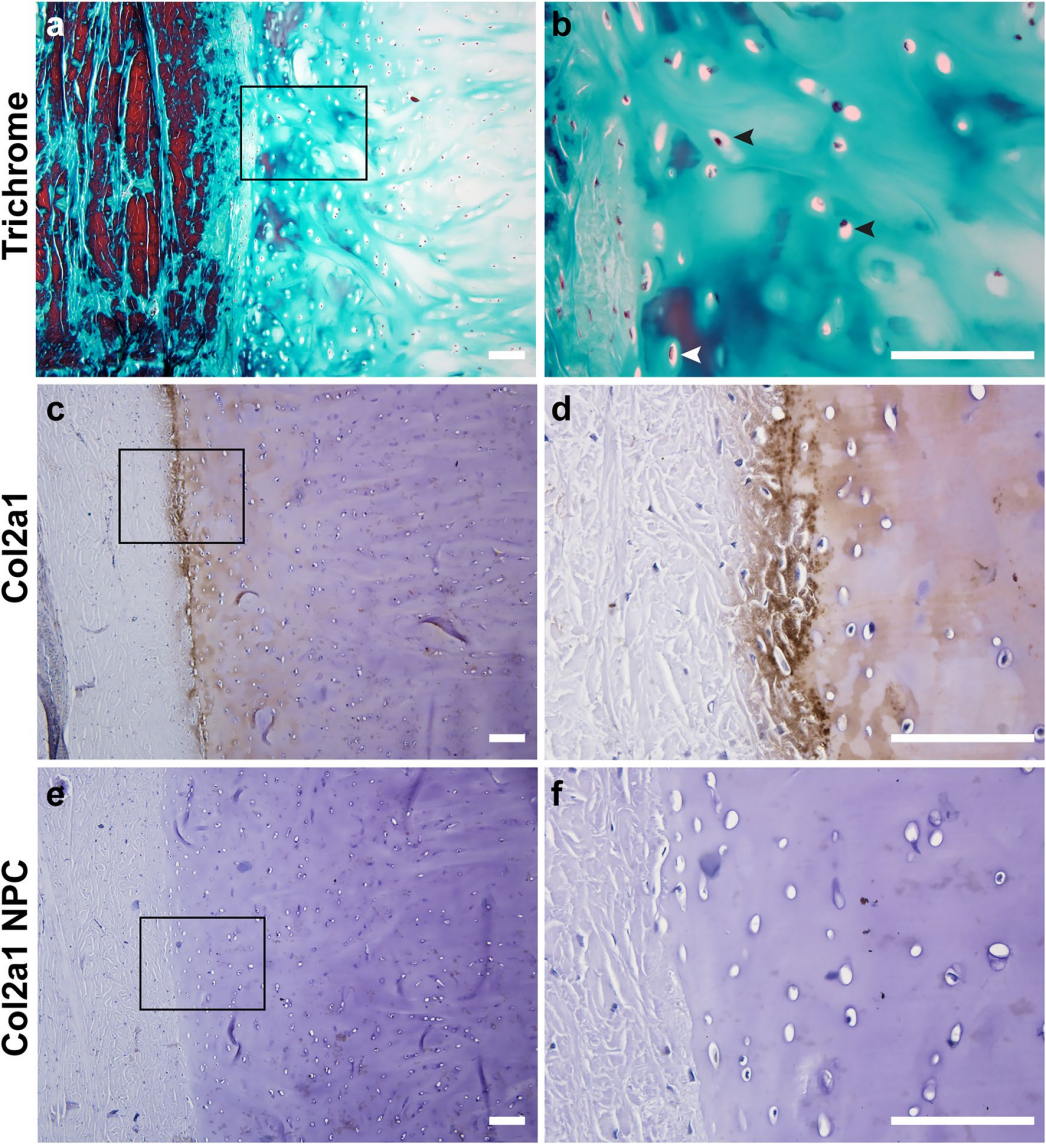
The endoskeleton of the regrown tail is composed of cartilage.Transverse sections stained with Gomori’s Trichrome (**a**,**b**) or subjected to IHC with an anti-collagen type II antibody and counterstained with hematoxylin (**c**–**f**). Tissue morphology is cartilage-like, with extensive ECM surrounding round cells in lacunae (black arrowheads), and lacking both vasculature and nerves. Smaller, denser chondrocytes (white arrowhead) are located at the interface between cartilage and the surrounding connective tissue. Images are representative and scale bars are 100 μm.

Histological examination of the endoskeletal structure confirmed it was composed of cartilage. First, Gomori’s trichrome staining of the tissue showed an avascular, collagen-rich extracellular matrix (ECM) that was sparsely populated with large, round chondrocytes embedded in lacunae (Fig. 6a,b black arrowheads). Chondrocytes closer to the interface of cartilage and the surrounding connective tissue were smaller and denser, (Fig. 6b white arrowhead). IHC using a broad species reactive antibody against collagen type II (COL2A1) identified a region that stained positive for this cartilage-specific protein^92^ (Fig. 6c,d). This area demarcated the cartilage from the overlying connective tissue. This antibody has been previously validated in reptiles^93^. Additionally, control sections with no primary antibody showed little to no background staining (Fig. 6e,f).

### The regrown tail segment lacks skeletal muscle and is comprised of a highly vascular, innervated network of collagen

**Figure 7:**
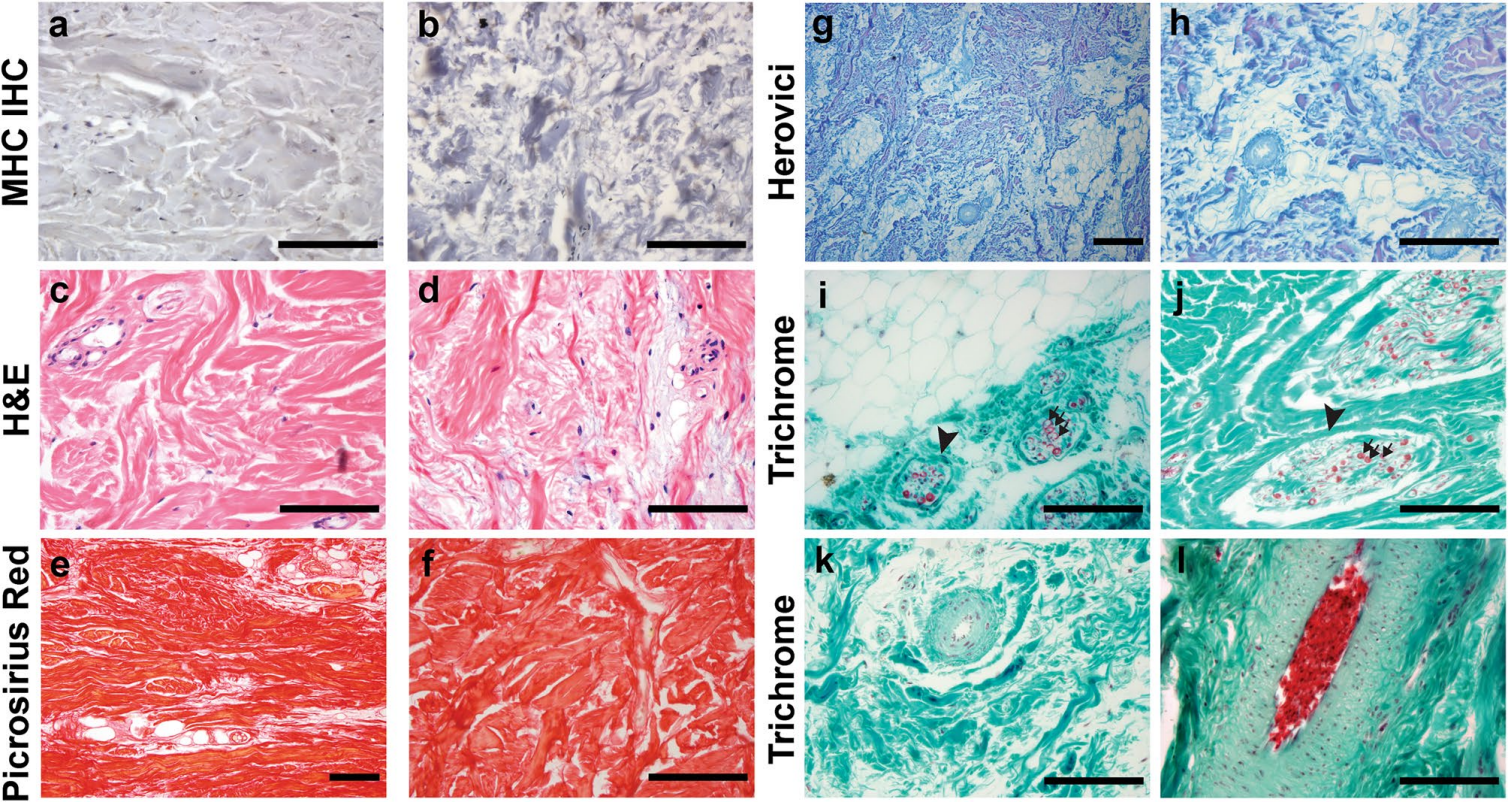
The regrown tail lacks skeletal muscle and is rich in collagen, adipose tissue, blood vessels, and axons. Staining with anti-MHC antibody and H&E shows that there is no skeletal muscle present in the regrown tail (**a**–**d**). Instead, the tissue is comprised of a dense network of collagen-based fibers that stain red with Picrosirius stain, and purple or blue with Herovici’s preparation, depending on whether type I or type III collagen is present (**e**–**h**). Pockets of adipose tissue and nerve bundles (black arrowheads) comprised of descending axons (black arrows) encased in a perineurium populated the tissue as well (**i**,**j**). Blood vessels of various sizes lined with smooth muscle and occasionally filled with erythrocytes were identified (**k**,**l**). Images are representative and scale bars are 100 μm.

Dissections revealed the regrown tail segment lacked skeletal muscle and immunostaining with an antibody recognizing MHC, a muscle-specific cell marker, confirmed this finding (Fig. 7a,b compare to Fig. 3e,f). A veterinary biopsy of one regrown tail segment indicated that there was excessive dermal collagen (Supplementary Data S3). Histological examination of other regrown tails corroborated this report and showed a dense network of irregular, fibrous connective tissue that was sparsely populated with mononucleated cells (Fig. 7c,d). The network of interlaced fibers stained red with Picrosirius Red dye, strongly suggesting it was collagen-based (Fig. 7e,f). Herovici’s polychrome, which distinguishes between different types of collagen, further showed that the larger fibers were predominantly type I collagen, while the smaller fibers, particularly when surrounding various structures, were type III collagen (Fig. 7g,h). This was consistent among all specimens analyzed. Interestingly, A03 contained notable, large pockets of adipocytes, which can be identified by their distinct lack of staining, and large, round appearance with eccentrically located nuclei (Fig. 7i).

The regrown tail was rich in axons, as well as blood vessels of varying size. Nerve bundles were often in close proximity to one another and can be morphologically distinguished in histological preparations as axons enclosed by a sheath of connective tissue (Fig. 7i,j). Given the lack of skeletal muscle in the regrown tail, we predict that these peripheral nerves are involved in sensory perception and not motor function. Blood vessels were also identified based on their distinct features, such as the presence of a lumen lined with endothelial cells and occasionally, smooth muscle (Fig. 7k). Within the lumen of the larger blood vessels we found erythrocytes, which in reptiles are eliptically-shaped, with a centrally located nucleus^94^ (Fig. 7l). Together, these data suggest that alligators exhibit some ability for regrowth, which may be dependent on the intrinsic properties of different tissue types.

## Discussion

**Figure 8:**
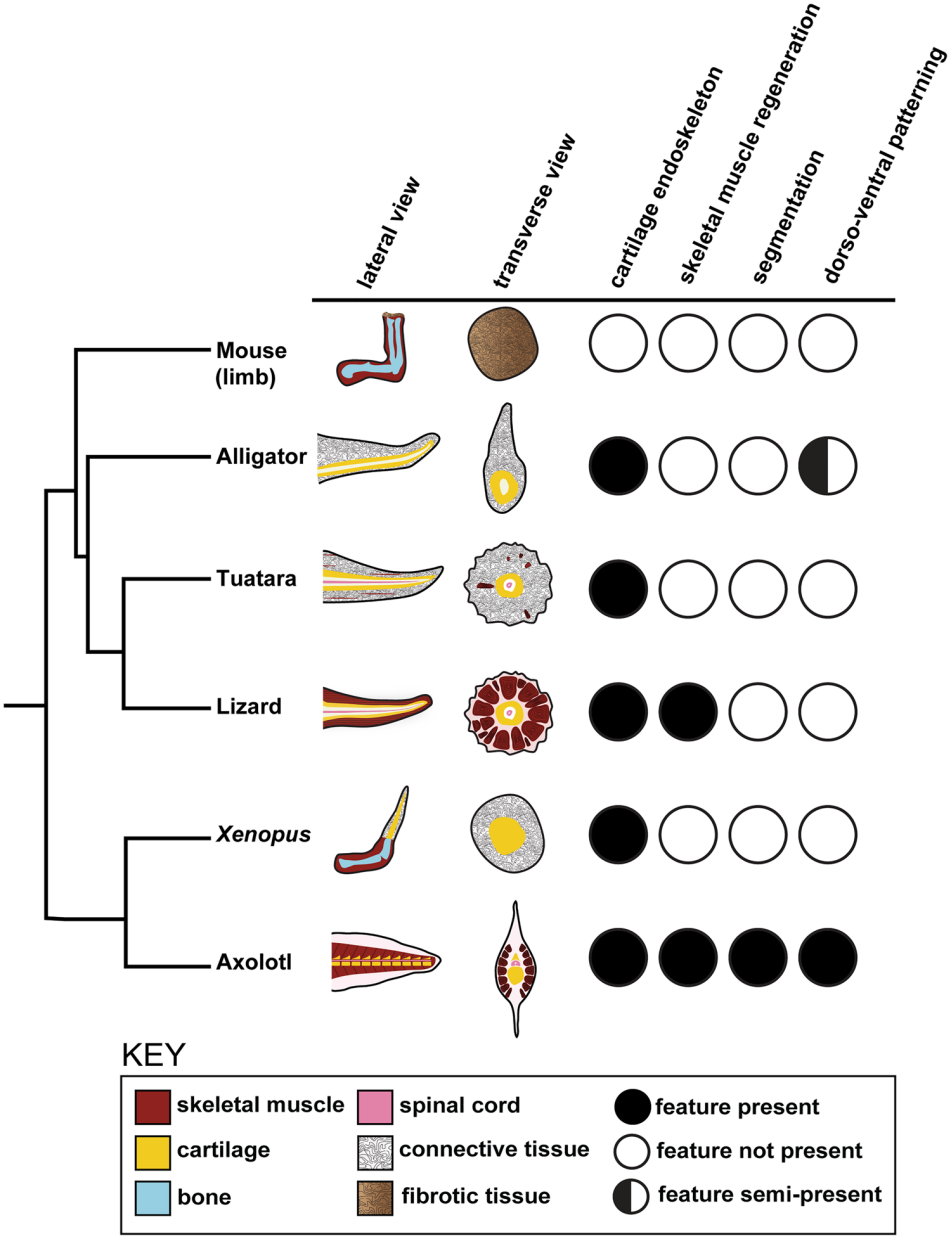
Anatomical similarities and differences between species capable of appendage regrowth or regeneration. Lateral and transverse schematics of regenerated limbs or tail in alligator*,* lizard, axolotl*,* and *Xenopus*, as well as wound repair in the mouse, are shown on the left. Conserved or divergent features of appendage regeneration or repair relative to the alligator regenerated tail is shown on the right.

In this study we show that tail wound repair in wild, juvenile American alligators (*Alligator mississippiensis*) is coupled with regrowth, opening up opportunities for comparative studies among vertebrates. Our data demonstrate that alligators can regrow their tails following substantial loss of the posterior tail segment, as well as after partial injury. Variation in regrown tail length may be due to sex, age, or environment as reptiles are exotherms. It is anticipated that tail repair with regrowth in the alligator is a prolonged process. For example, in the black caiman, tail regrowth following conspecific amputation of the posterior tail segment was observed up to 15 months^70^ and 18 months in another study^71^. While the aforementioned reports suggest that crocodilians are capable of tail regrowth, it is unknown how or when alligators analyzed in this study lost their tails. Tail loss in crocodilians can be caused by male-male intraspecific aggression or cannibalism of juveniles by larger individuals^95–98^, which may have been the case in the specimens characterized in this study, although other injuries caused by boat motor blades are also possible^99,100^. Alternatively, the observed abnormal morphology may have been caused by repair from injuries sustained during embryonic development or congenital birth defects related to axial patterning. However, we hypothesize that the abnormal morphology described here is a result of regrowth following post-hatching tail injury, rather than congenital birth defects. If developmental anomalies had occurred, we would expect to see defects in multiple caudal vertebrae and/or agenesis of vertebrae^101,102^. However, we observed fissure planes and an abnormal spinous process in only the caudal vertebrae nearest the junction site, whereas the rest of the vertebrae were comparable in morphology to those in the original, intact tail. We have identified a distinct pattern of tail repair and regrowth in the alligator that demonstrates features in common with lepidosaurian and amphibian appendage regeneration, as well as mammalian wound healing (Fig. 8). However, this study captures only the end product of wound repair and/or regrowth. Future studies monitoring temporal changes in morphology following a controlled amputation would be necessary to determine the developmental mechanisms regulating this process. Such studies in American alligators are challenging, given that the species is listed as threatened, and thus protected, under the US Endangered Species Act.

There are many similarities between tail regeneration in lizards and the tuatara and alligator tail repair with regrowth. The regenerated tail in lizards and the tuatara features an unsegmented, cartilaginous tube that extends along the proximo-distal axis, replacing the segmented vertebrae^1–4^. The regrown alligator endoskeleton was also composed of cartilage, and was structurally similar to the lizard, including radial symmetry and randomly distributed foramina. Ventral positioning of the cartilage endoskeleton indicated that some patterning information was retained during tail repair with regrowth. Although this is reminiscent of the ventral cartilage rod in salamander regenerated tails, the alligator regrown endoskeleton does not transition to reform articulated caudal vertebrae nor re-establish dorso-ventral organization^103–106^. Instead the cartilaginous endoskeleton remains persistently unaltered, which is likely a conserved feature of non-avian reptile tail regrowth. This is further supported by paleontological evidence of the Jurassic marine crocodile, *Steneosaurus bollensis*, which exhibited an ossified cartilage rod that measured 12 cm in length, suggesting that tail repair with regrowth is an ancestral trait of modern crocodilians^107,108^.

However, there are also striking differences between tail regrowth in the lizard versus the alligator. Lizards can regenerate tails with elongated, axial skeletal muscle groups that are radially organized and are capable of flexion^3^. Surprisingly, skeletal muscle was entirely absent from the regrown alligator tail, which indicates the inability to flex that segment. The alligator tail is the main effector of propulsive thrust for locomotion and predatory behaviors^109–112^. Thus, we hypothesize that the anterior, original tail is sufficient for locomotion in animals that survived and were found healthy after tail injury. As described above, *M. caudofemoralis* was uninjured in all individuals analyzed. This powerful, hypaxial muscle is responsible for retraction of the hind limb, which permits lateral displacement of the alligator tail and may enhance muscle force^85,113,114^. Although the regrown tail may not be involved in locomotive performance, repair with regrowth of the tail could help maximize propulsive surface area. The regrown alligator tail segment lacked skeletal muscle but featured a dense network of irregular, fibrous connective tissue including type I and type III collagens. Mammals, which have a very limited capacity for regeneration, produce a higher ratio of collagen type I than type III (6:1) collagen during scar formation^115^. The replacement of normal tissue with an overproduction of extracellular matrix components is characteristic of scar tissue^116^.

The absence of skeletal muscle in the regrown alligator tail segment is also observed in the regenerated tuatara tail^44^ and in the *Xenopus* frog forelimb^117^ following injury. Moreover, amputation of the *Xenopus* limb post-metamorphosis (stage 53) results in the formation of a hypomorphic spike composed of an unsegmented cartilage rod that lacks associated skeletal muscle and is surrounded by connective tissue^47,117–121^. The regenerated froglet limb also exhibits evidence of dorso-ventral axis patterning similar to the regrown alligator tail. In sexually mature male frogs with regenerated spikes, nuptial pad tissue consistently reforms on the ventral surface^122,123^. For both the *Xenopus* limb and the alligator tail, we would expect that there is regrowth of the sensory nervous system but that evolutionary selective pressures have not required skeletal muscle and motor nervous system regeneration for active appendage flexion.

Comparative studies have been instrumental in addressing why some animals can regenerate complex, multi-tissue structures while others, such as birds and mammals, have lost this ability. One major hypothesis as to why regenerative competency was lost is the evolution of an adaptive immune system. For example, amputation in salamanders induces weak inflammatory responses^124^, and these animals can regrow structures nearly identical to the original. On the other hand, *Xenopus* skin and limb regenerative abilities are reduced as development proceeds; this is concurrent with the maturation of the immune system, including an increase in T cells^47,125,126^. However, alligators and lizards have both adaptive and innate immune systems as complex as mammals and birds. Moreover, alligators are known to mount strong, broad acting immunological responses in addition to the documentation of T and B cells^127–131^. Furthermore, the initial injury and immunological response is critical in non-avian reptile regeneration^132,133^. Previous transcriptomic analyses in the green anole lizard revealed many genes involved in the innate and adaptive immune response as well as genes enriched for extracellular matrix remodeling, wound epidermis formation, and re-innervation, which together inhibit fibrosis and initiate the regenerative program^134^. However, in our study, all individuals analyzed in this study were either juveniles or sub-adults, raising the question of whether adult alligators exhibit the same repair with regrowth capacity after tail amputation.

The alligator also provides a model to examine regrowth and its trade-offs at a much larger scale than the lizard. Body size is associated with changes in life history, metabolic rates, and energy allocation^135^. Regenerating an appendage is an energetically expensive process and it has been shown that in some lizards, tail regeneration decreased the overall growth rate^136,137^. Smaller lizards such as the anole regenerate tail segments on the order of a few centimeters and weighing a few grams, whereas the regrown tails even in the juvenile alligators exceeded 10 cm and 100 g. In the larger alligator, the designation of resources towards regenerating tissues such as skeletal muscle, which has high metabolic activity, may be more costly to either developmental growth or reproduction.

The ancestor of both crocodilians and birds arose approximately 250 million years near the end of the Permian, and the two lineages diverged afterwards during the early Triassic (> 245 mya)^138^. In the crocodilian lineage, there is paleontological evidence of tail regrowth in the Jurassic marine crocodile *S. bollensis*^107,108^. In contrast, paleontological evidence in the avian lineage is limited to descriptions of bone healing of caudal vertebrae from presumptive trauma in non-avian dinosaurs^74,139–144^, leaving open the question of when the ability for repair with regrowth was lost. Further analysis of tail regrowth in alligators could identify the molecular and cellular processes that are conserved regenerative mechanisms in lizards, salamanders and other vertebrates.

## Supplementary information

Supplementary information.

Supplementary Video 1.

Supplementary Video 2.

Supplementary Video 3.

Supplementary Data 1.

Supplementary Data 2.

Supplementary Data 3.

## Data Availability

All data generated or analysed during this study are included in this published article (and its Supplementary Information files).
