# Supplementary material for: Anatomical and histological analyses reveal that tail repair is coupled with regrowth in wild-caught, juvenile American alligators (Alligator mississippiensis)
Source: Sci Rep. 2020 Nov 18;10:20122. doi: 10.1038/s41598-020-77052-8 (PMC7674433; doi:10.1038/s41598-020-77052-8)
Supplement: Supplementary file 1 — Supplementary information. [file 41598_2020_77052_MOESM1_ESM.pdf]

Supplemental Information for

Anatomical and histological analyses reveal that tail repair is coupled with regrowth in wild-caught, juvenile American alligators (*Alligator mississippiensis*)

Cindy Xu[1], Joanna Palade[1], Rebecca E. Fisher[1,2], Cameron I. Smith[1], Andrew R. Clark[1], Samuel Sampson[1], Russell Bourgeois[3], Alan Rawls[1], Ruth M. Elsey[4], Jeanne Wilson-Rawls[1*], Kenro Kusumi[1,*]

[1] School of Life Sciences, Arizona State University, Tempe, AZ 85287, USA

[2] Department of Basic Medical Sciences, University of Arizona College of Medicine-Phoenix, Phoenix, AZ 85004, USA

[3] Russell Bourgeois, Jeanerette, Louisiana 70544, USA

[4] Rockefeller Wildlife Refuge, Louisiana Department of Wildlife and Fisheries, Grand Chenier, LA 70643, USA

[*] Corresponding authors

Jeanne.Wilson-Rawls@asu.edu

Kenro.Kusumi@asu.edu

P.O. Box 874501, Tempe, AZ 85287, USA

Table of Contents

Supplementary Information **Page**

Supplementary Figure S1.......Caudal vertebrae are associated with a single row of scales 1

Supplementary Video S1 ....3D reconstruction of an alligator regrown endoskeleton 2

Supplementary Video S2 ....Transverse 2D serial sections of a regrown alligator tail.............3

Supplementary Video S3 ....Lateral 2D serial sections of a regrown alligator tail...................4

Supplementary Data S1..........Photographs of tail regrowth in alligators...................................5

Supplementary Data S2..........Photographs of wound healing and fibrosis in alligators.............6

Supplementary Data S3..........Regrown tissue biopsy report for specimen A04.........................7

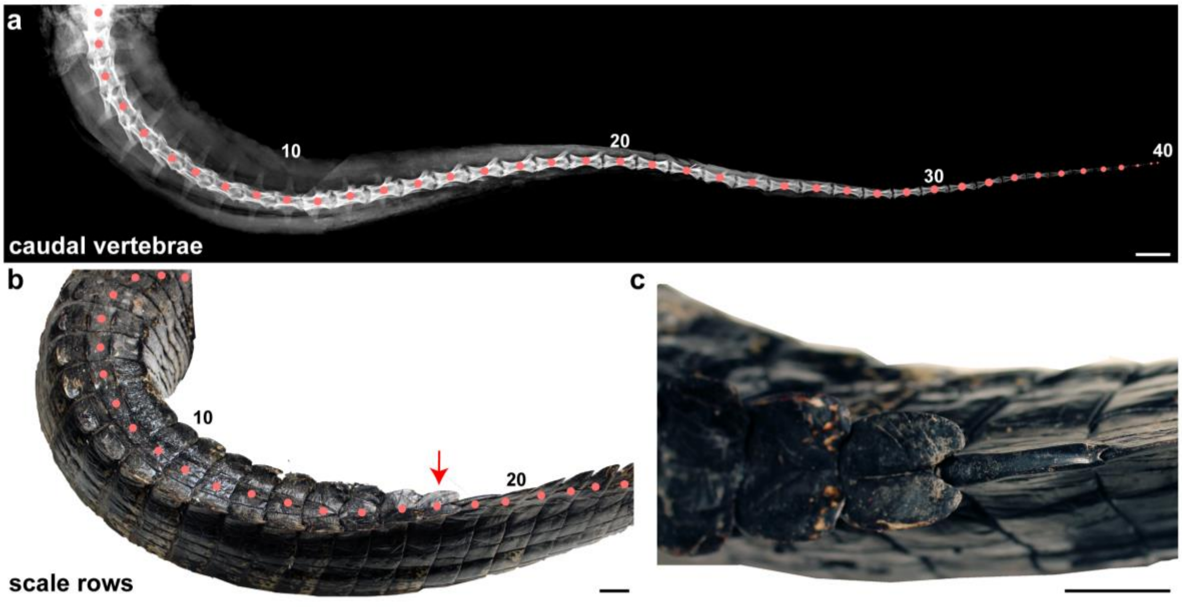

Supplementary Fig. S1 Caudal vertebrae are associated with a single row of scales. Lateral radiograph of an original alligator tail with a total of 40 caudal vertebrae (a). Each caudal vertebra (Ca) is associated with a single transverse row of scales and paired dorsal scutes terminate at Ca 18 (b, c). Individual caudal vertebrae and scale rows are represented by red dots. The red arrow indicates the segment at which paired dorsal scutes terminate. Scale bars: 2.5 cm

Supplementary Video S1 3D reconstruction of an alligator regrown endoskeleton. The total volume of the regrown endoskeleton (A01) measures 1270 cm^3. [Consult supplementary files]

Supplementary Video S2 Transverse 2D serial sections of a regrown alligator tail. [Consult supplementary files]

Supplementary Video S3 Lateral 2D serial sections of a regrown alligator tail. [Consult supplementary files]

Supplementary Data S1 Photographs of tail regrowth in alligators. Images courtesy of Louisiana Department of Wildlife and Fisheries. [Consult supplementary files]

Supplementary Data S2 Photographs of wound repair and fibrosis in alligators. Images courtesy of Louisiana Department of Wildlife and Fisheries. [Consult supplementary files]

Supplementary Data S3 Regrown tissue biopsy report for specimen A04. [Consult supplementary files]
