# Supplementary figures and images for: Anatomical and histological analyses reveal that tail repair is coupled with regrowth in wild-caught, juvenile American alligators (Alligator mississippiensis)
Source: Sci Rep. 2020 Nov 18;10:20122. doi: 10.1038/s41598-020-77052-8 (PMC7674433; doi:10.1038/s41598-020-77052-8)

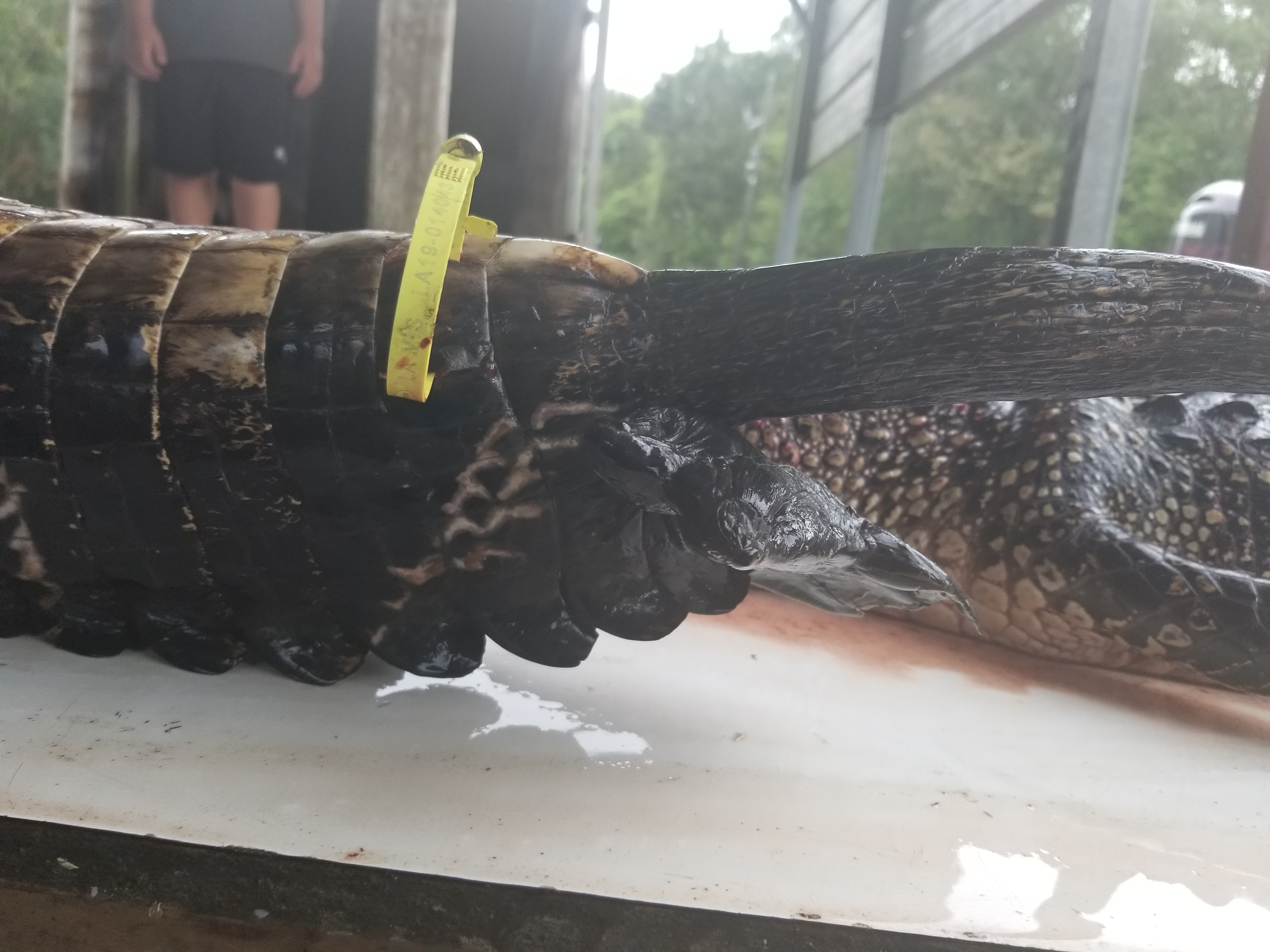

Supplement: Supplementary file 5 — Supplementary Data 1. [file 41598_2020_77052_MOESM5_ESM.zip › SData1/A05_RT/A05_image_1.jpg]

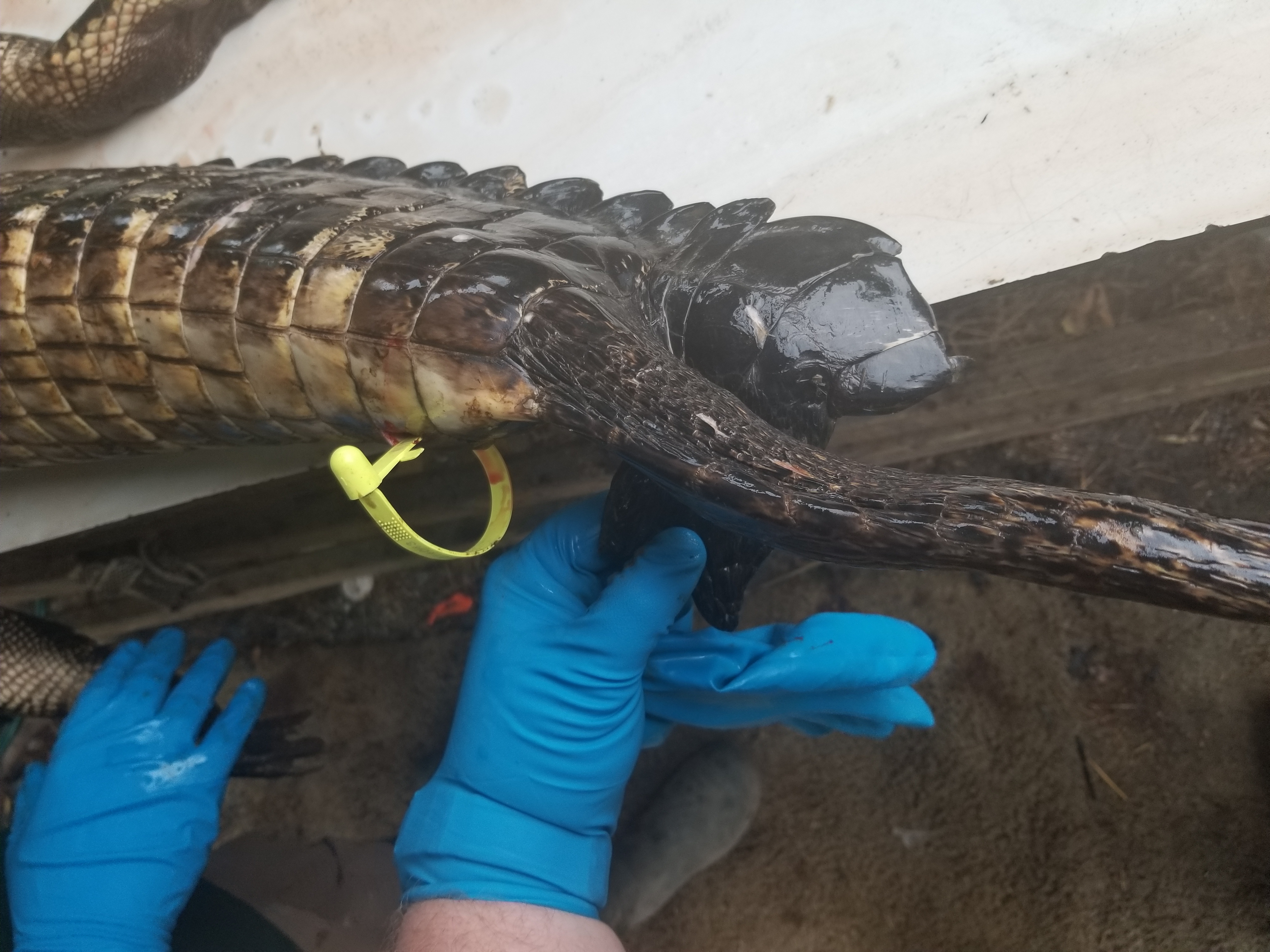

Supplement: Supplementary file 5 — Supplementary Data 1. [file 41598_2020_77052_MOESM5_ESM.zip › SData1/A05_RT/A05_image_2.jpg]

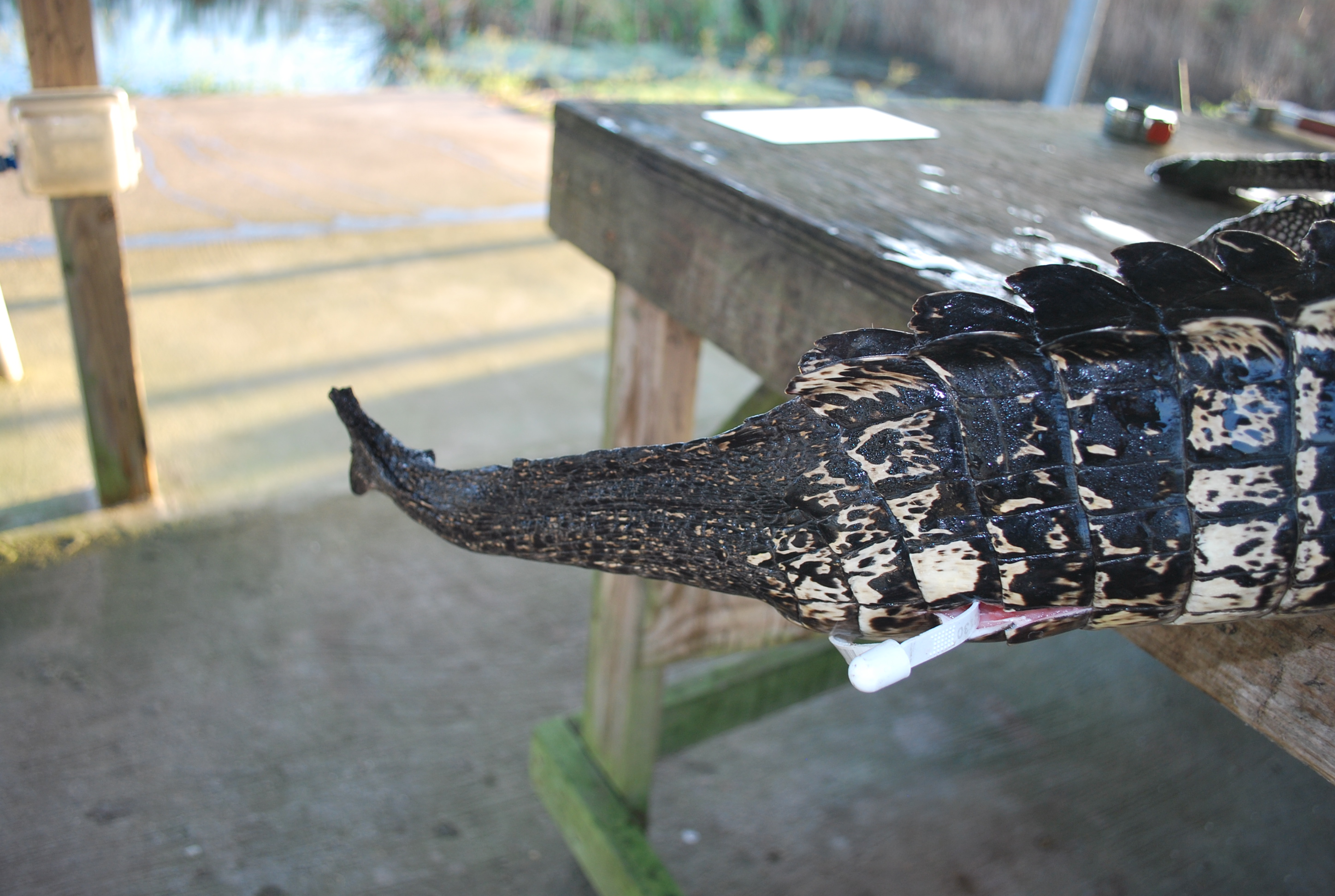

Supplement: Supplementary file 5 — Supplementary Data 1. [file 41598_2020_77052_MOESM5_ESM.zip › SData1/A01_F_RT/A01_F_image_3.JPG]

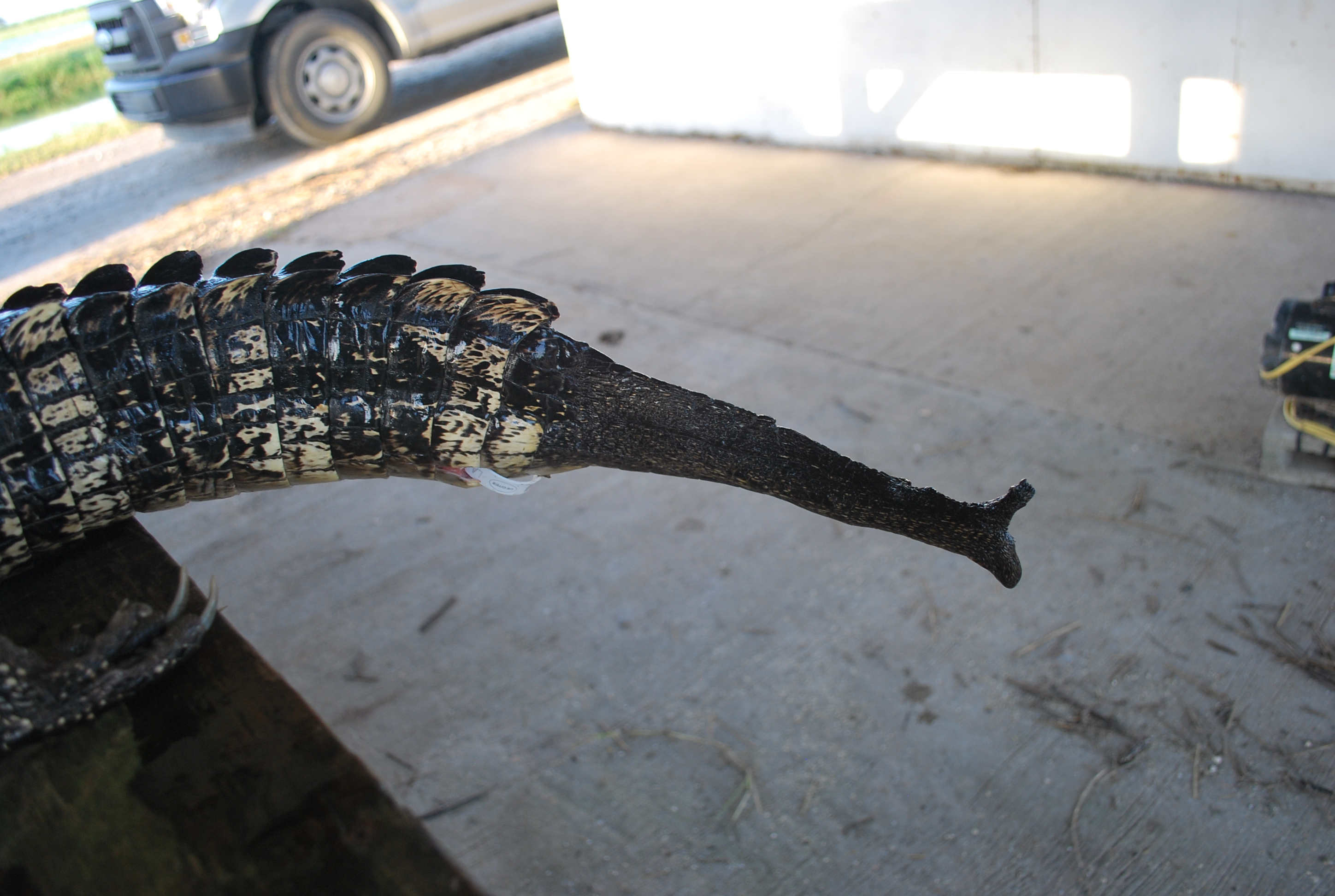

Supplement: Supplementary file 5 — Supplementary Data 1. [file 41598_2020_77052_MOESM5_ESM.zip › SData1/A01_F_RT/A01_F_image_2.JPG]

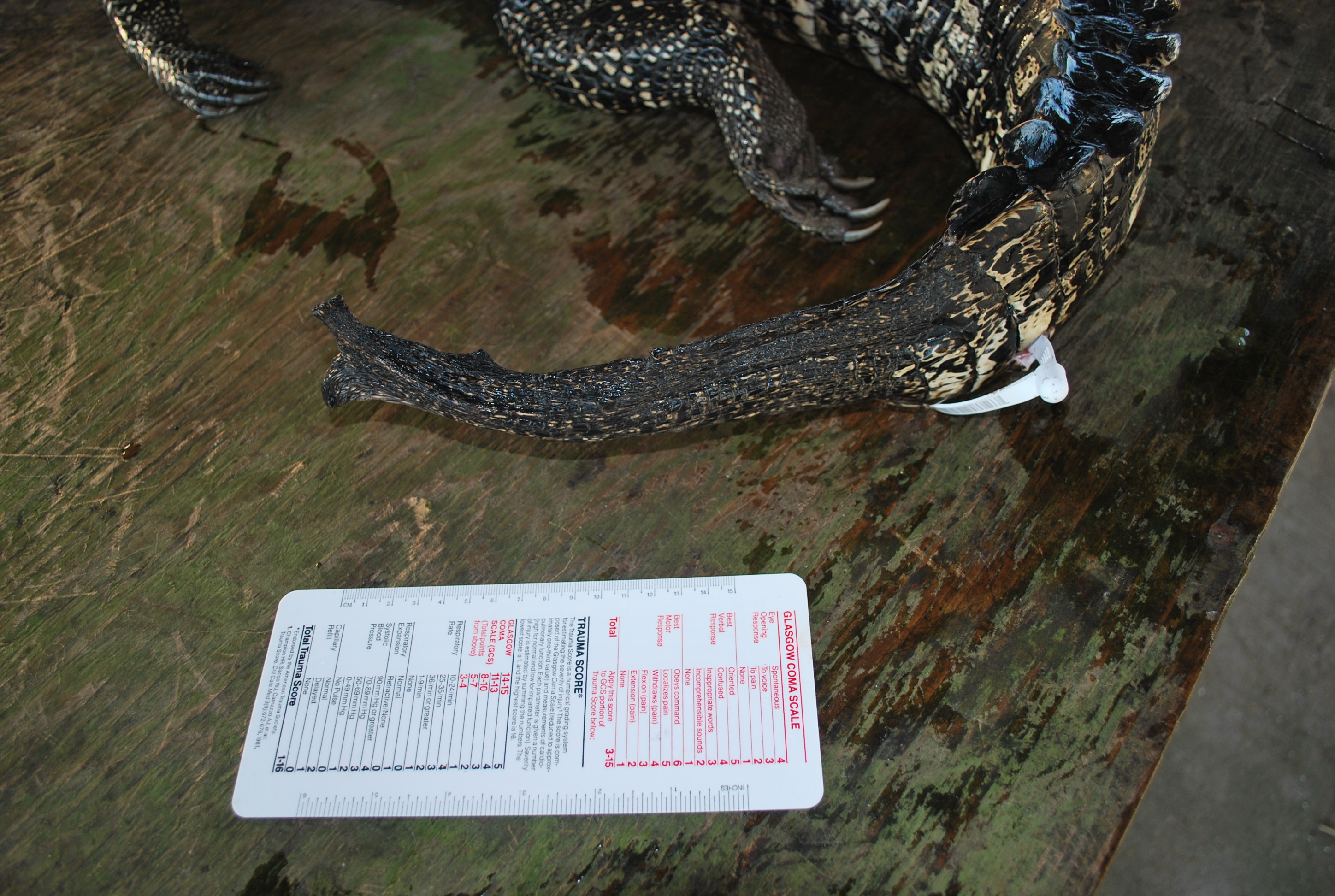

Supplement: Supplementary file 5 — Supplementary Data 1. [file 41598_2020_77052_MOESM5_ESM.zip › SData1/A01_F_RT/A01_F_image_1.JPG]

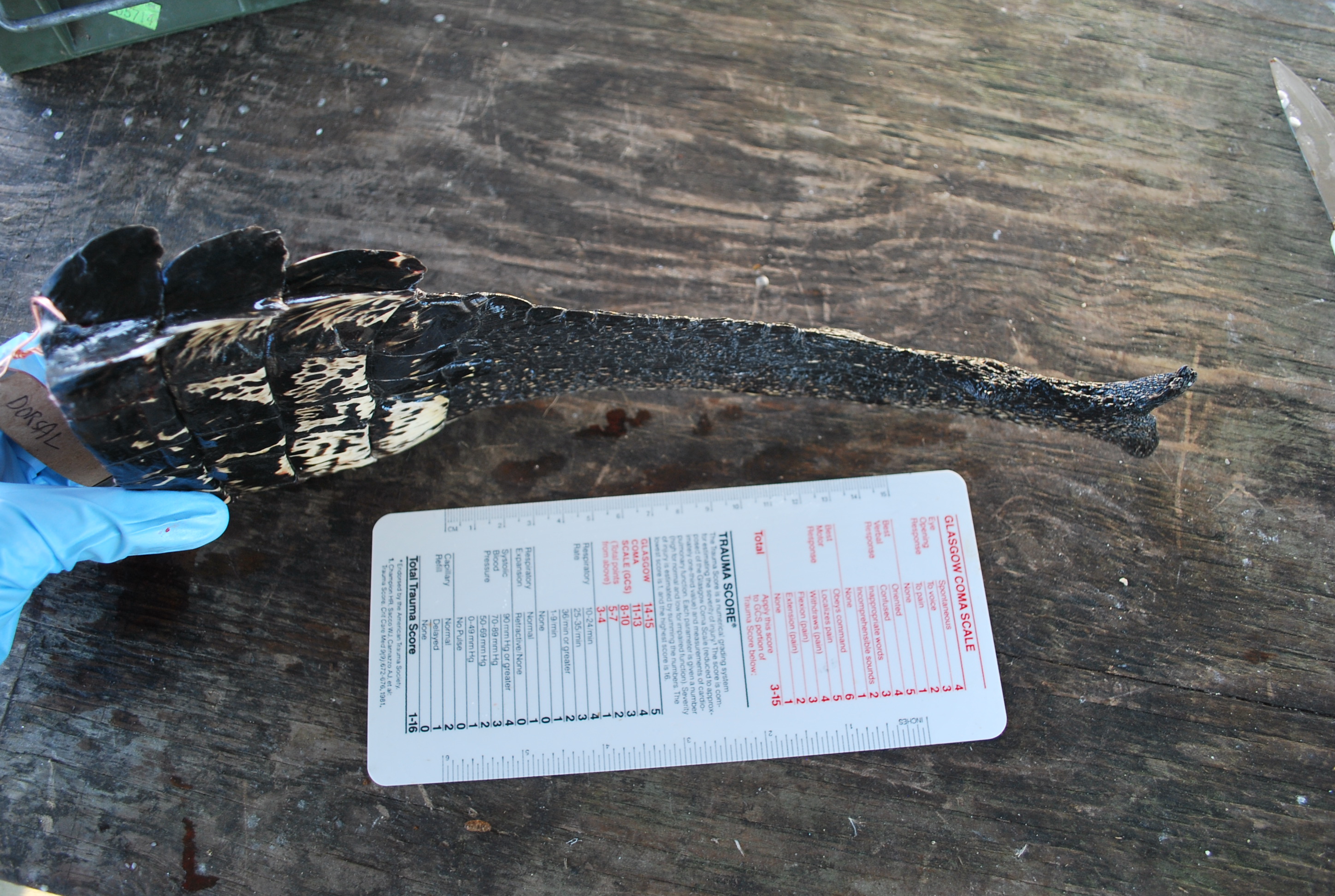

Supplement: Supplementary file 5 — Supplementary Data 1. [file 41598_2020_77052_MOESM5_ESM.zip › SData1/A01_F_RT/A01_F_image_5.JPG]

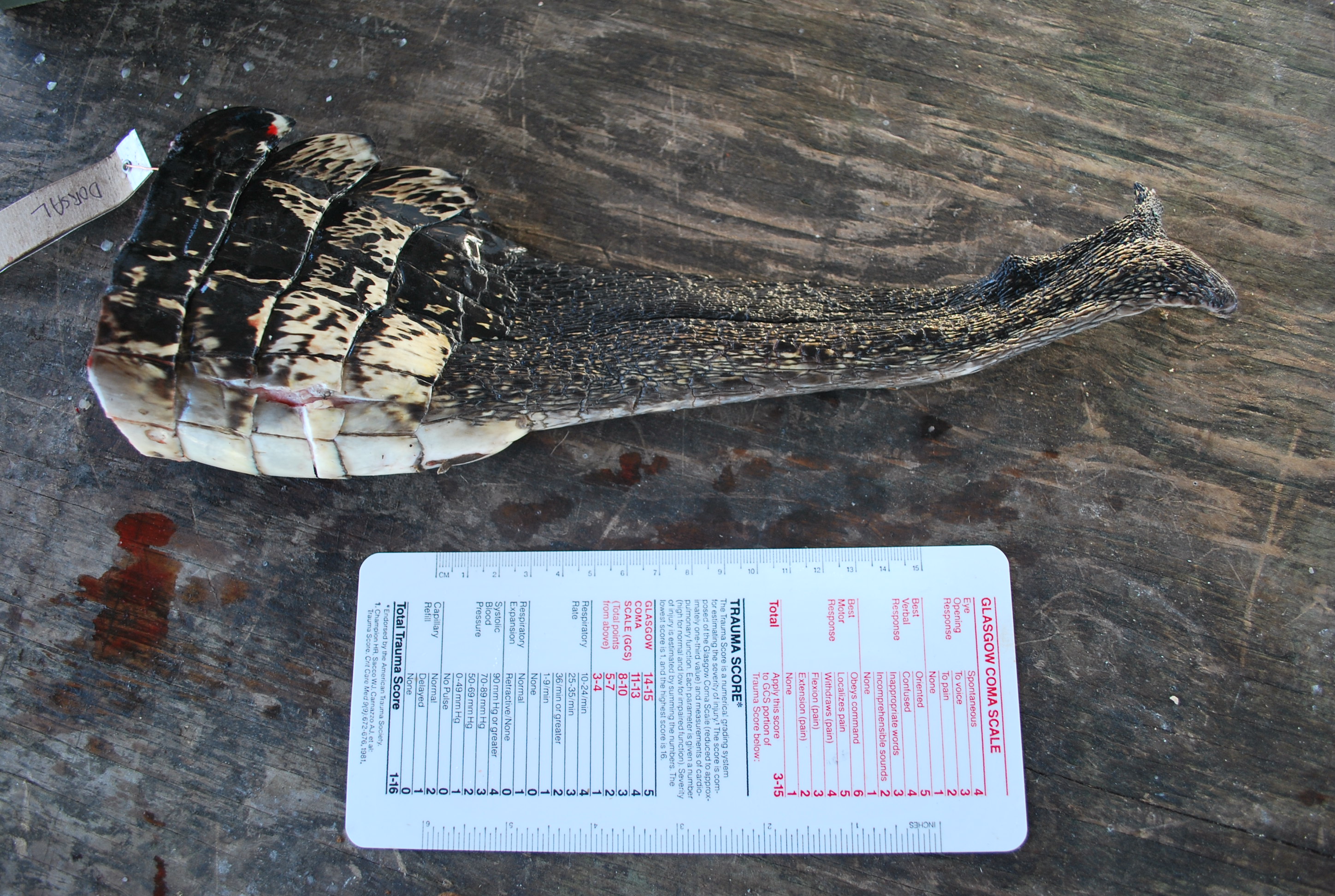

Supplement: Supplementary file 5 — Supplementary Data 1. [file 41598_2020_77052_MOESM5_ESM.zip › SData1/A01_F_RT/A01_F_image_4.JPG]

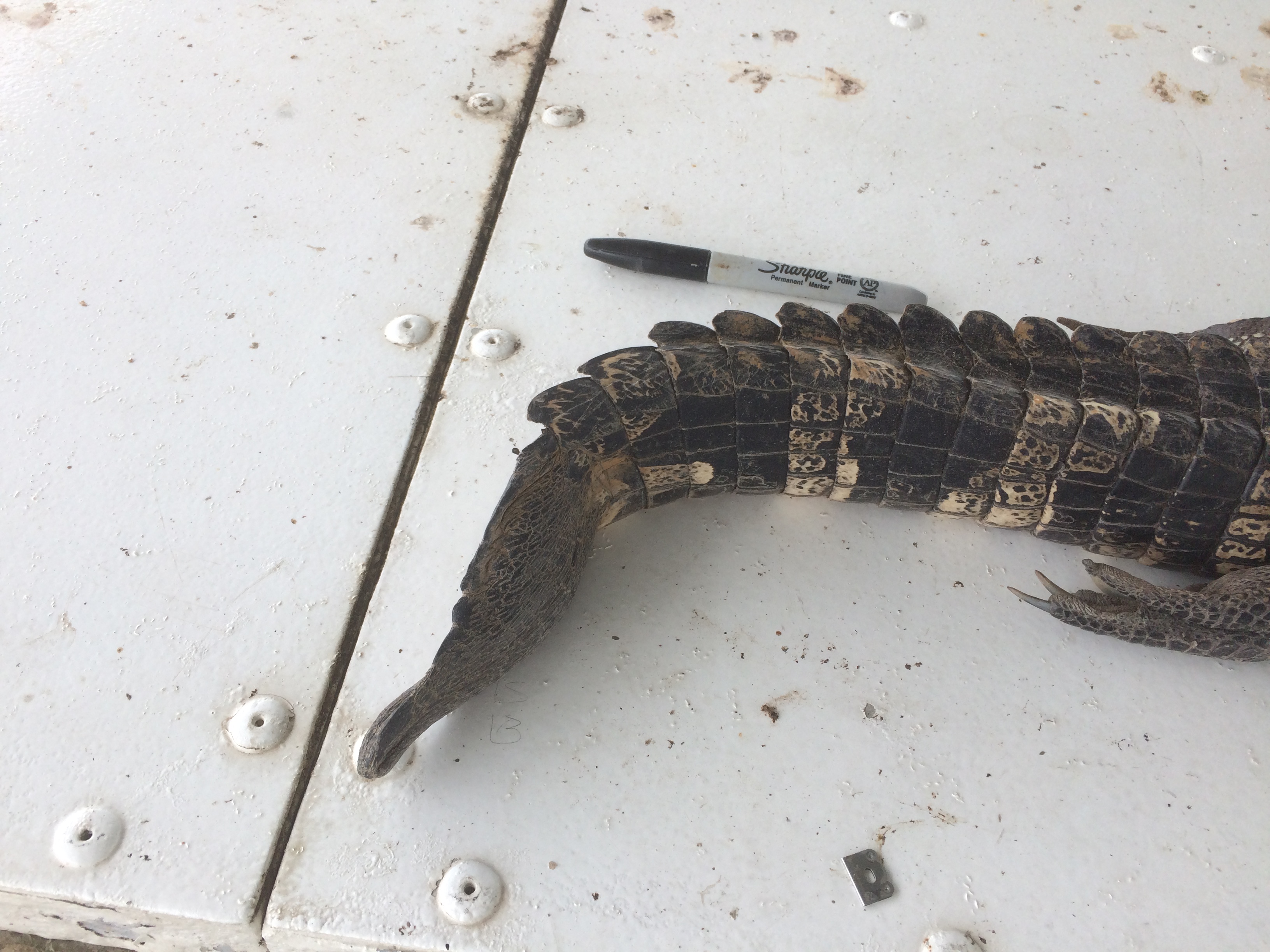

Supplement: Supplementary file 5 — Supplementary Data 1. [file 41598_2020_77052_MOESM5_ESM.zip › SData1/A03_F_RT/A03_F_image_1.JPG]

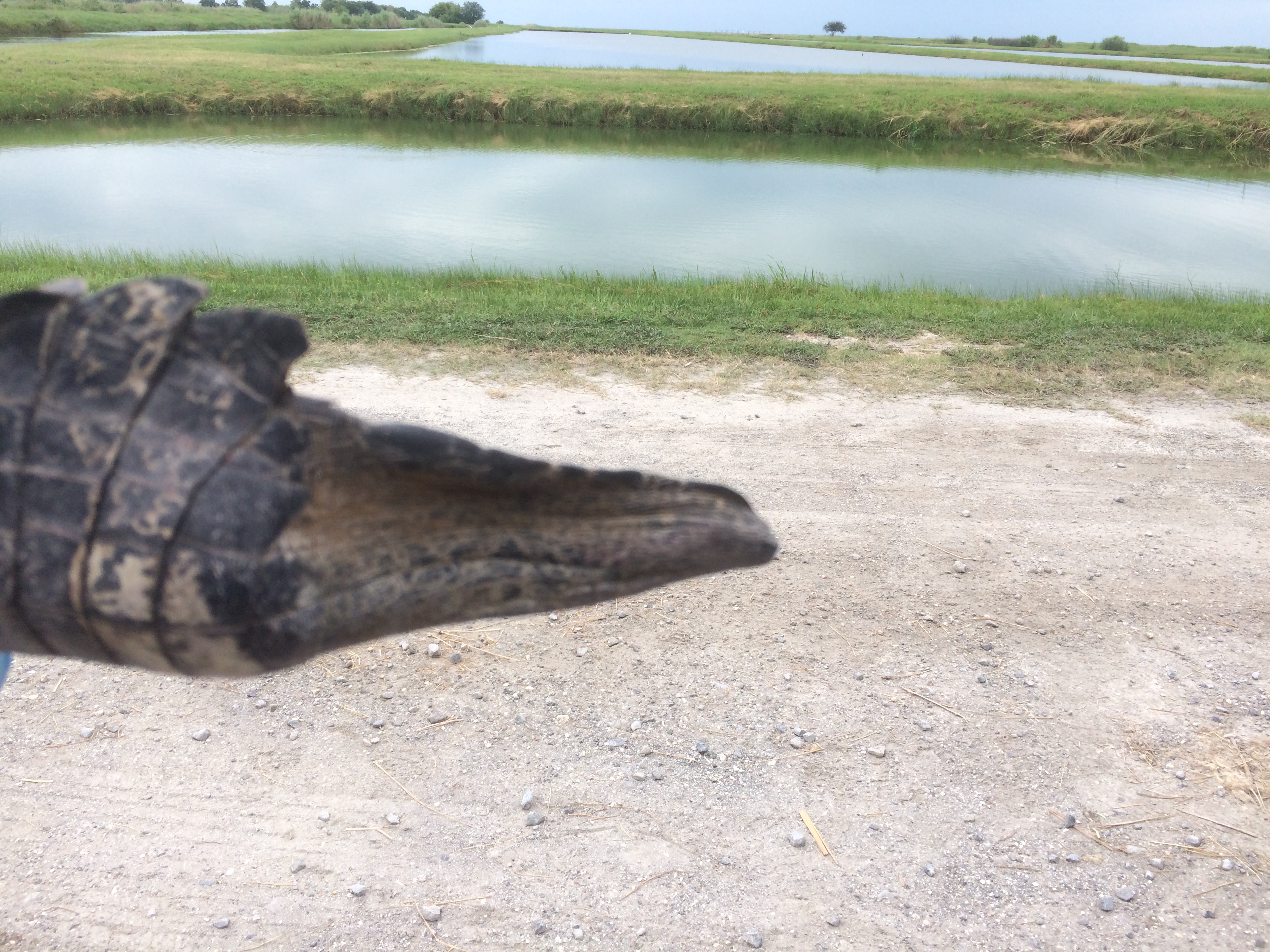

Supplement: Supplementary file 5 — Supplementary Data 1. [file 41598_2020_77052_MOESM5_ESM.zip › SData1/A03_F_RT/A03_F_image_2.JPG]

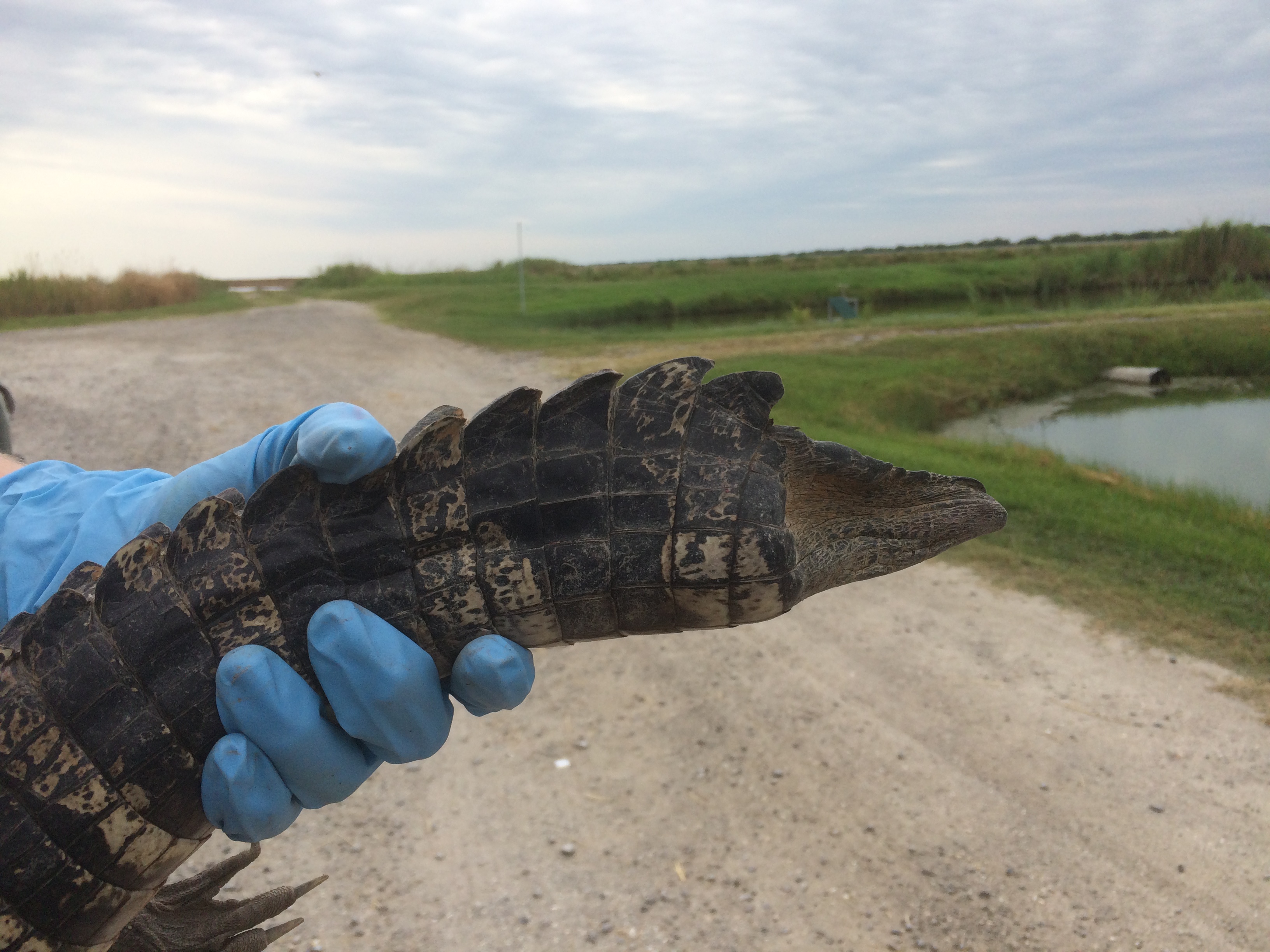

Supplement: Supplementary file 5 — Supplementary Data 1. [file 41598_2020_77052_MOESM5_ESM.zip › SData1/A03_F_RT/A03_F_image_3.JPG]

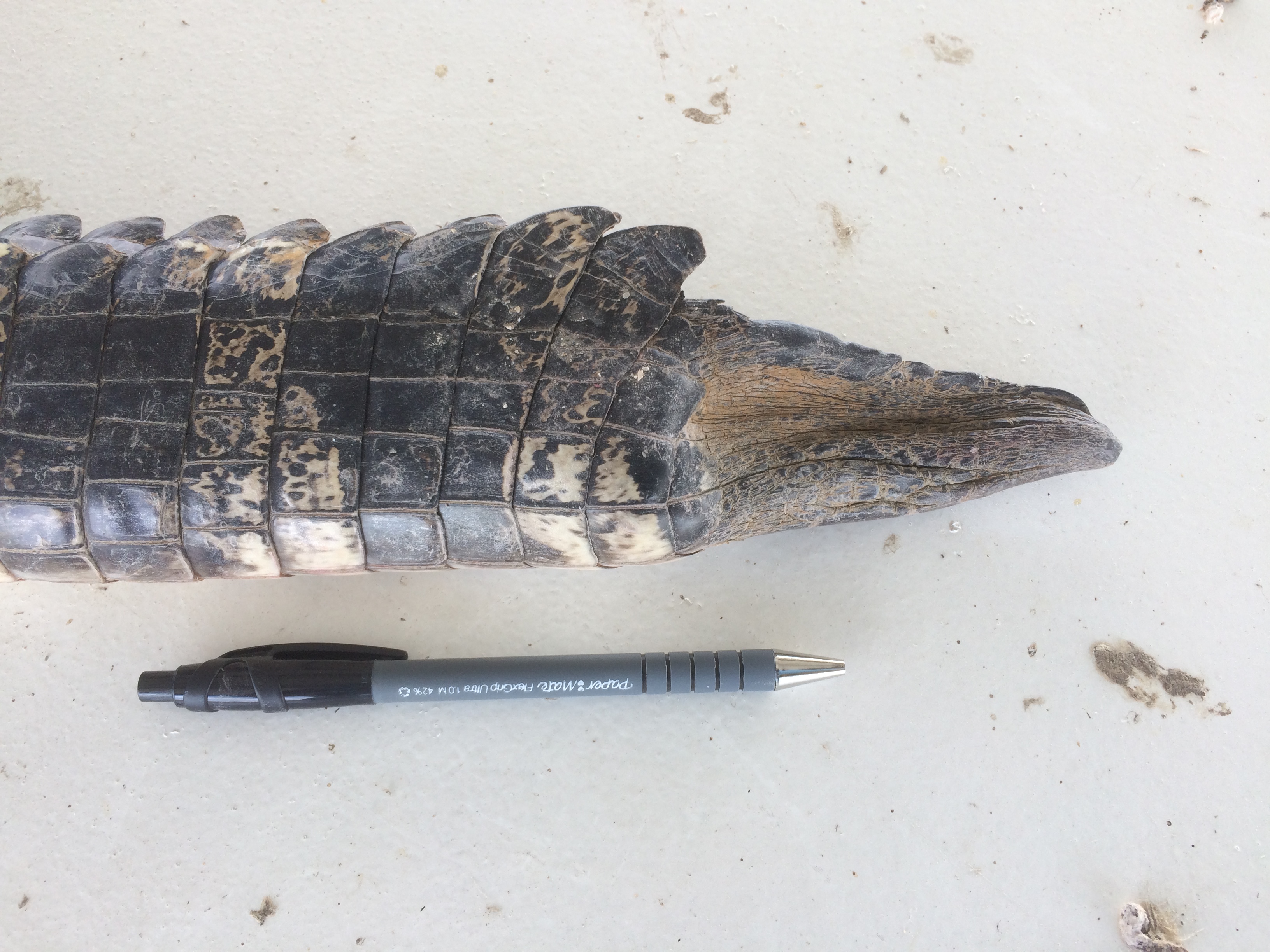

Supplement: Supplementary file 5 — Supplementary Data 1. [file 41598_2020_77052_MOESM5_ESM.zip › SData1/A03_F_RT/A03_F_image_4.JPG]

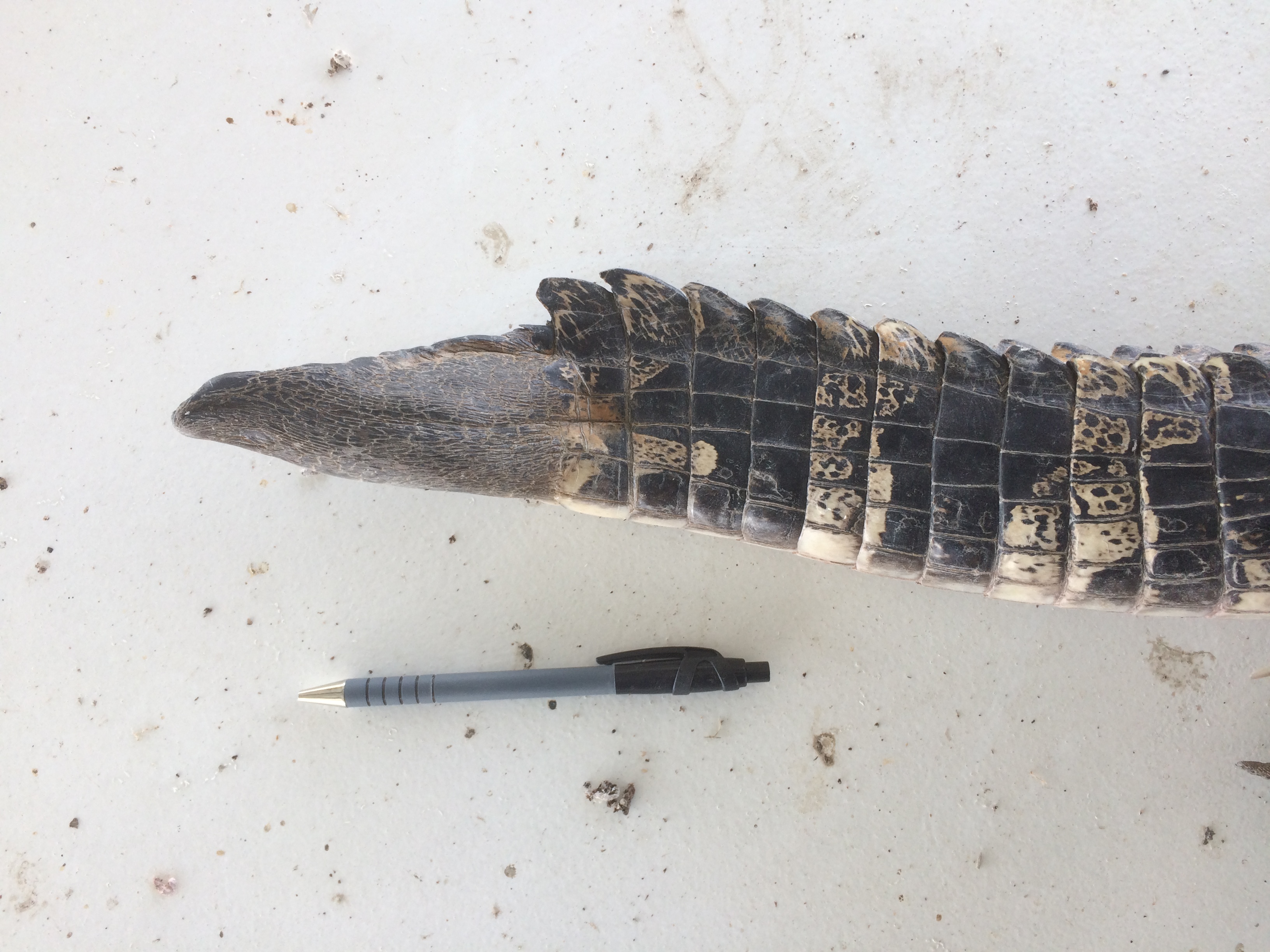

Supplement: Supplementary file 5 — Supplementary Data 1. [file 41598_2020_77052_MOESM5_ESM.zip › SData1/A03_F_RT/A03_F_image_5.JPG]

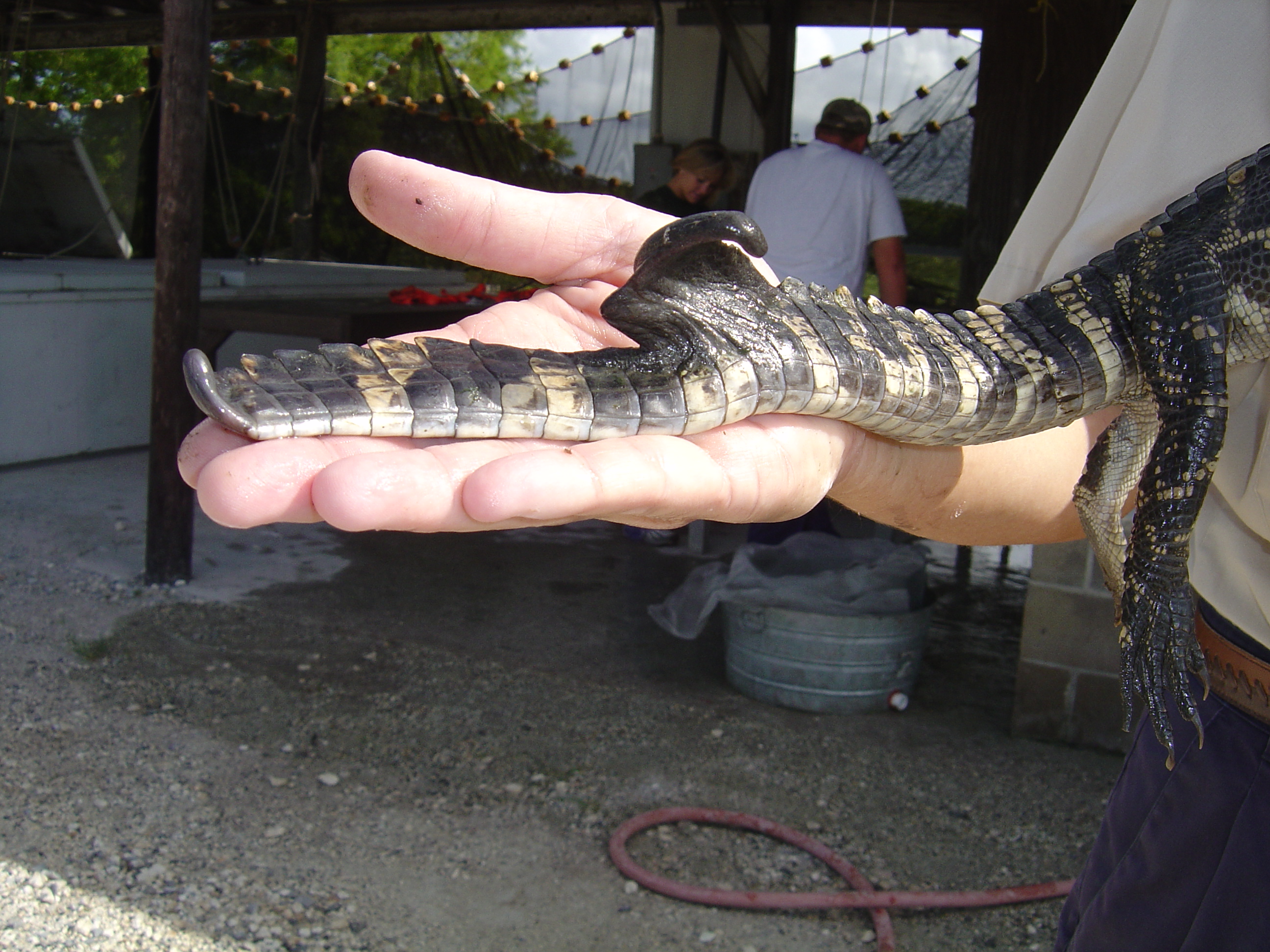

Supplement: Supplementary file 5 — Supplementary Data 1. [file 41598_2020_77052_MOESM5_ESM.zip › SData1/A04_M_RT/A04_M_image_6.JPG]

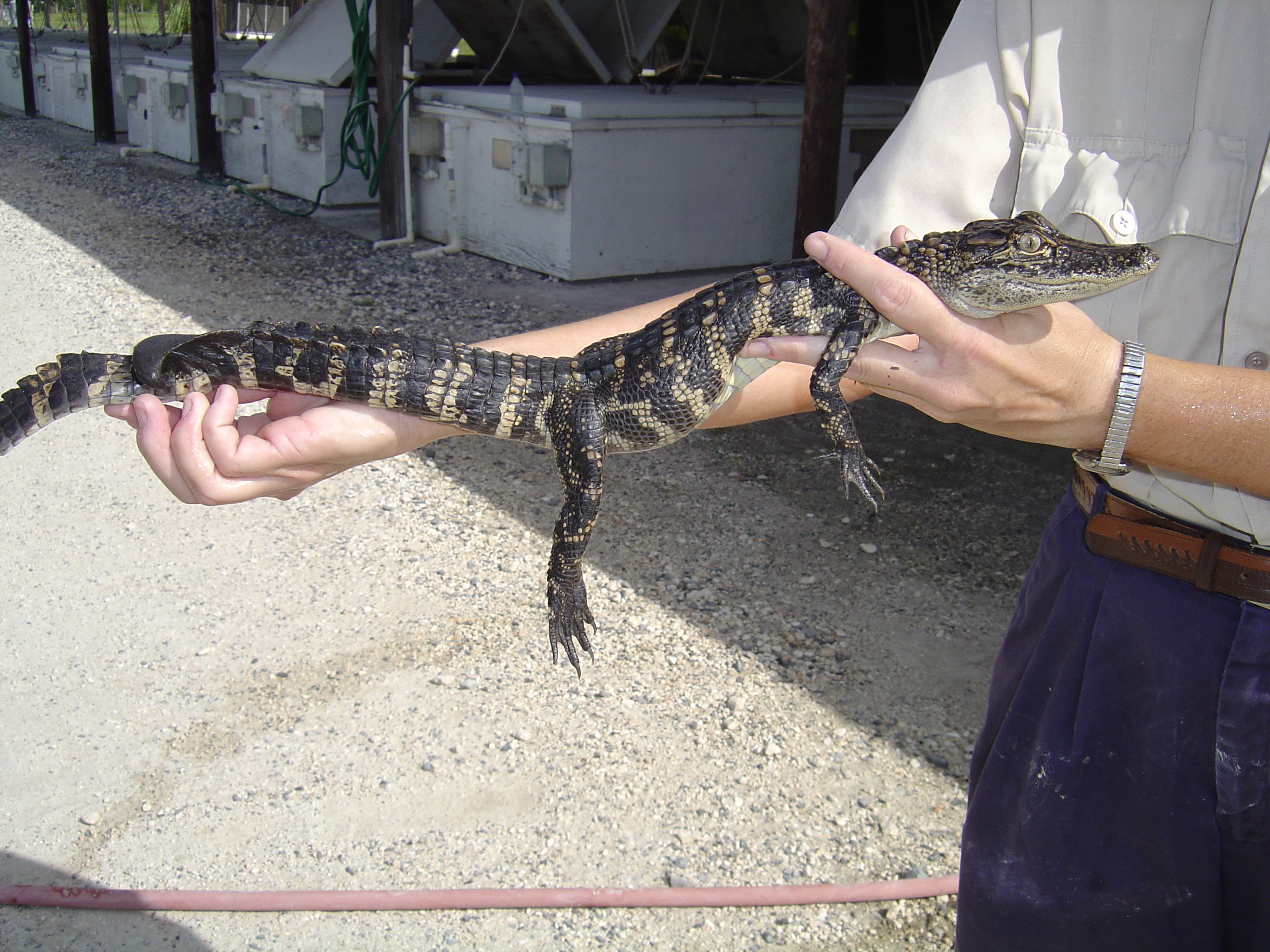

Supplement: Supplementary file 5 — Supplementary Data 1. [file 41598_2020_77052_MOESM5_ESM.zip › SData1/A04_M_RT/A04_M_image_7.JPG]

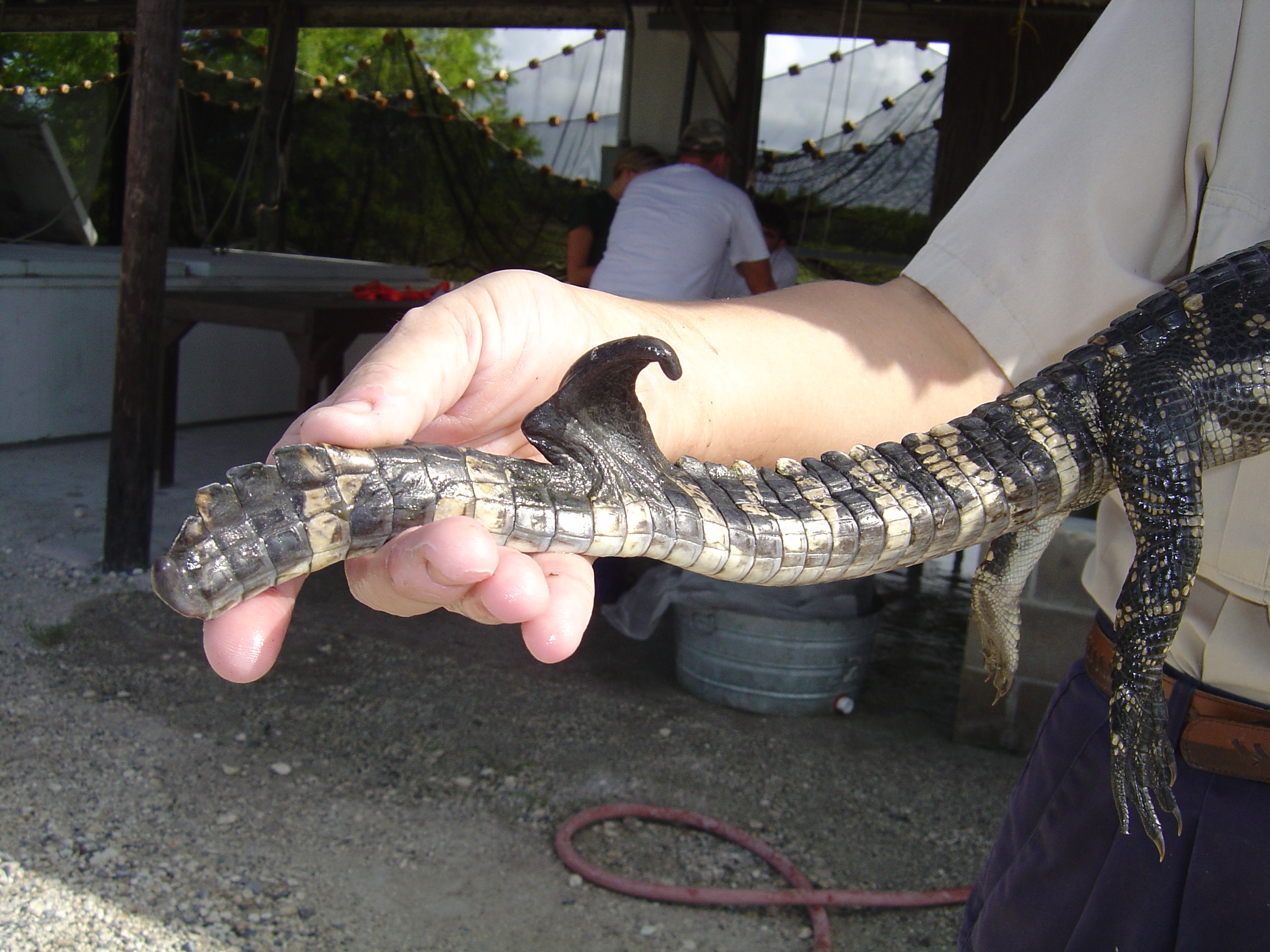

Supplement: Supplementary file 5 — Supplementary Data 1. [file 41598_2020_77052_MOESM5_ESM.zip › SData1/A04_M_RT/A04_M_image_5.JPG]

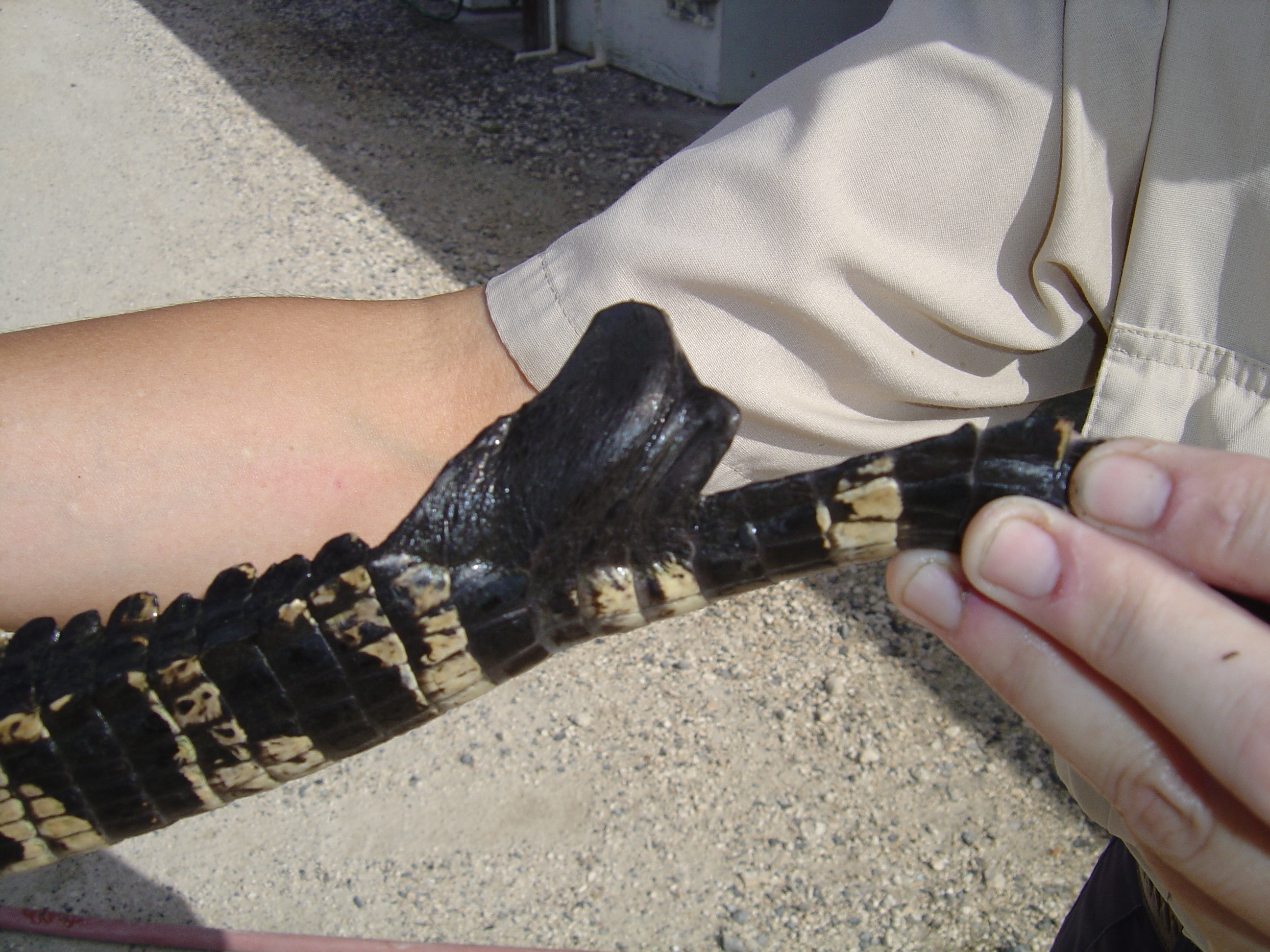

Supplement: Supplementary file 5 — Supplementary Data 1. [file 41598_2020_77052_MOESM5_ESM.zip › SData1/A04_M_RT/A04_M_image_4.JPG]

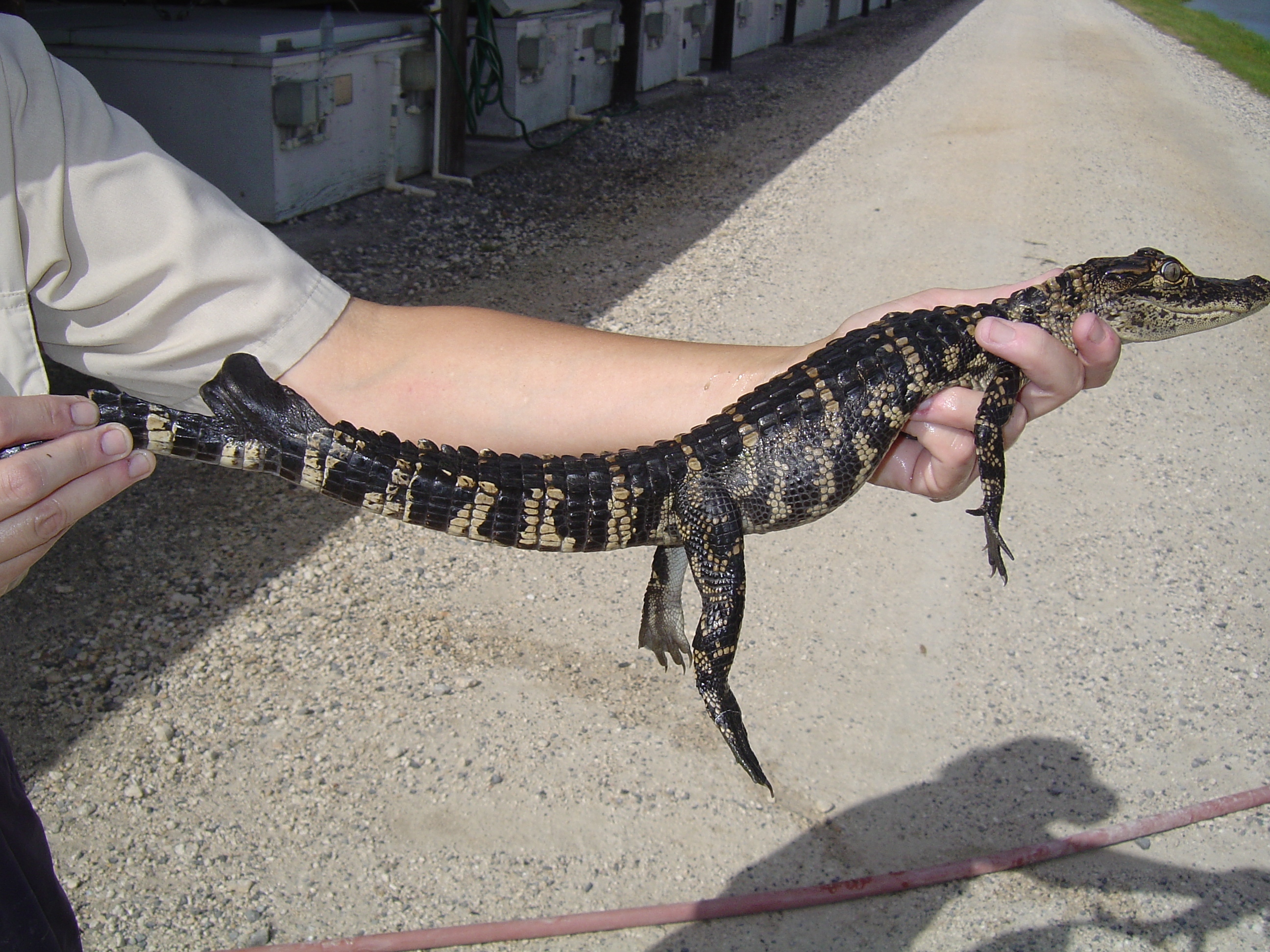

Supplement: Supplementary file 5 — Supplementary Data 1. [file 41598_2020_77052_MOESM5_ESM.zip › SData1/A04_M_RT/A04_M_image_1.JPG]

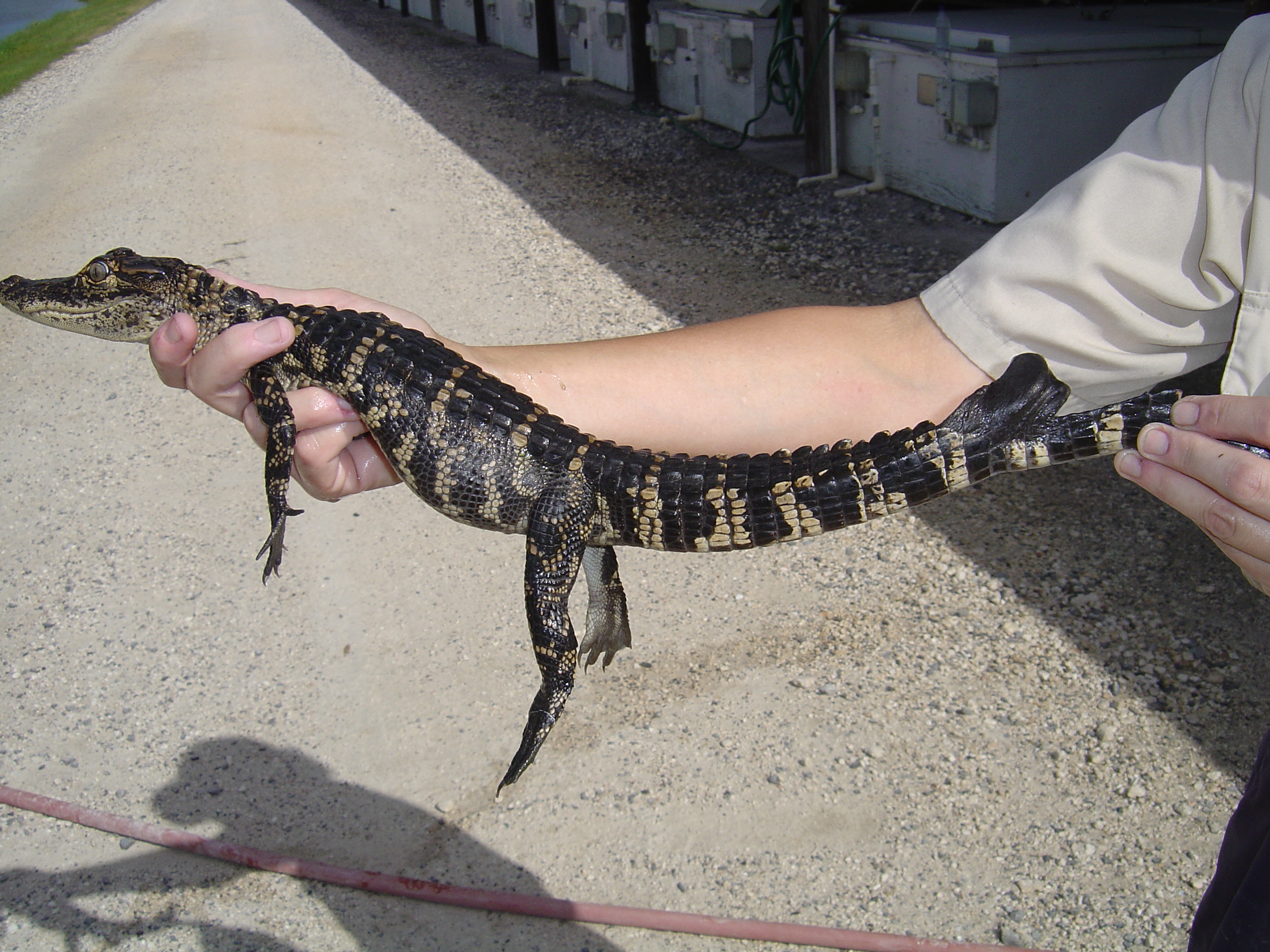

Supplement: Supplementary file 5 — Supplementary Data 1. [file 41598_2020_77052_MOESM5_ESM.zip › SData1/A04_M_RT/A04_M_image_3.jpg]

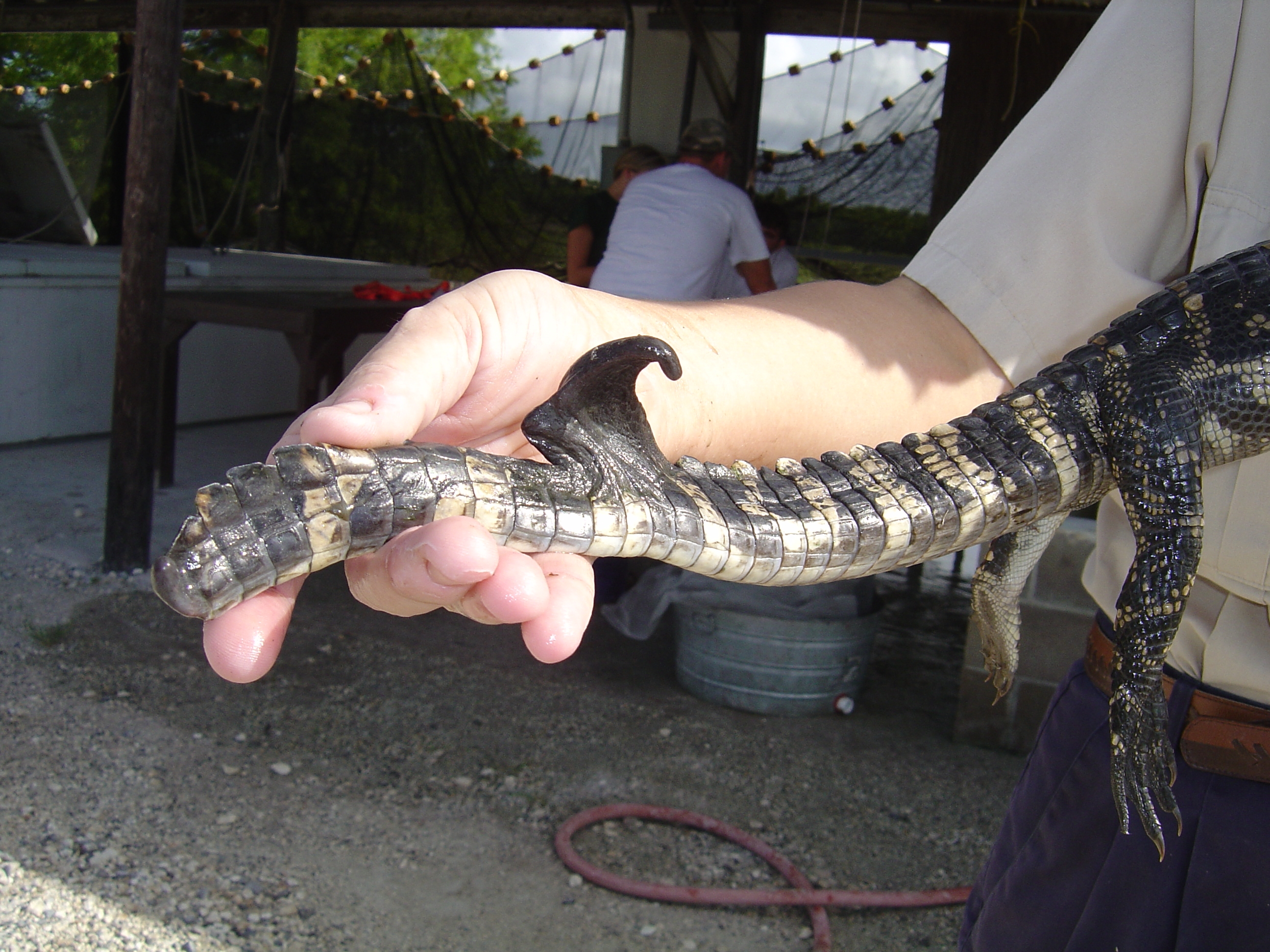

Supplement: Supplementary file 5 — Supplementary Data 1. [file 41598_2020_77052_MOESM5_ESM.zip › SData1/A04_M_RT/A04_M_image_2.JPG]

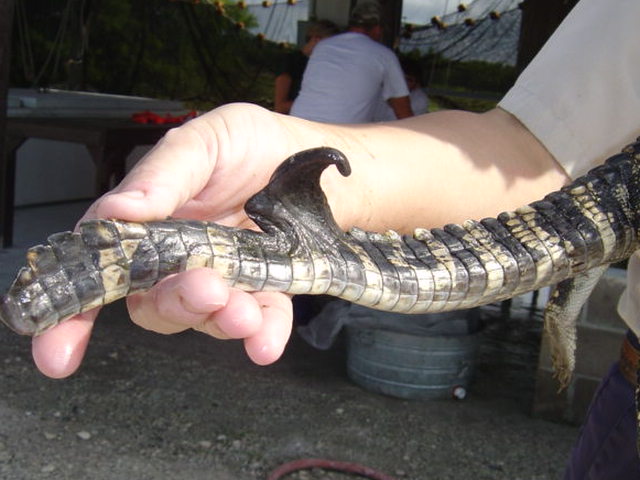

Supplement: Supplementary file 5 — Supplementary Data 1. [file 41598_2020_77052_MOESM5_ESM.zip › SData1/A04_M_RT/A04_M_image_9.JPG]

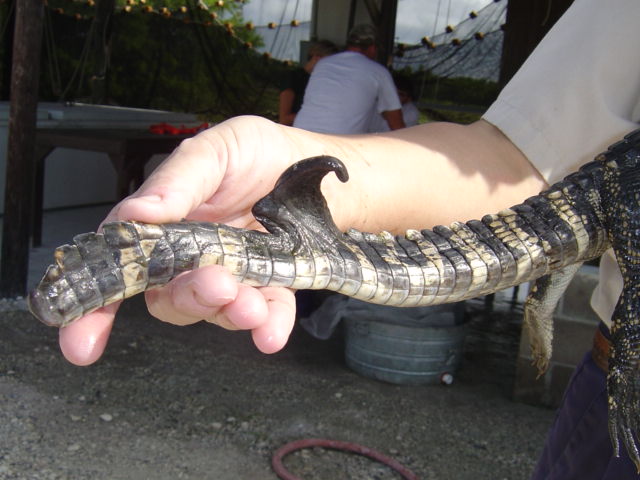

Supplement: Supplementary file 5 — Supplementary Data 1. [file 41598_2020_77052_MOESM5_ESM.zip › SData1/A04_M_RT/A04_M_image_8.JPG]

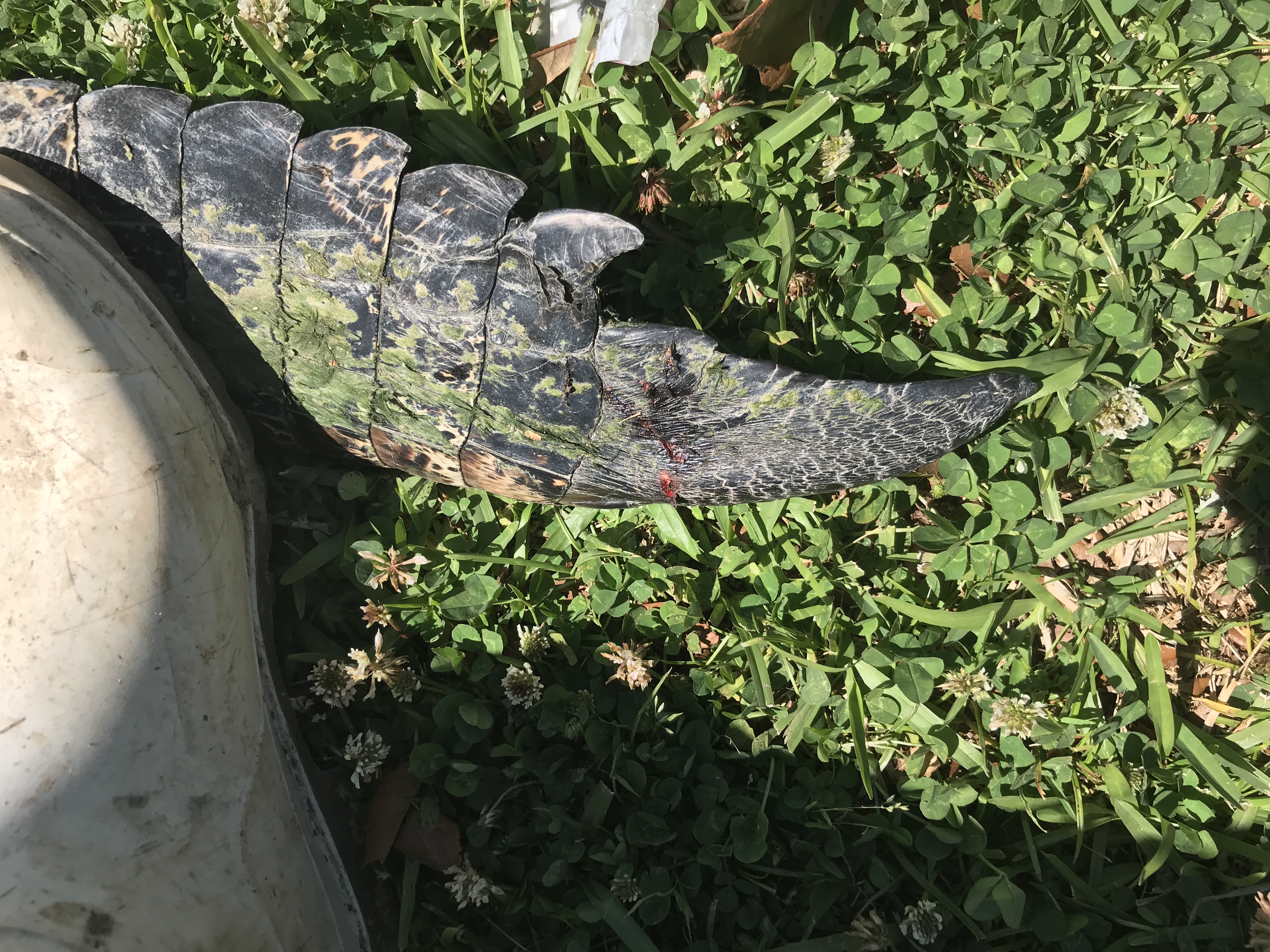

Supplement: Supplementary file 5 — Supplementary Data 1. [file 41598_2020_77052_MOESM5_ESM.zip › SData1/A02_M_RT/A02_M_image_1.jpeg]

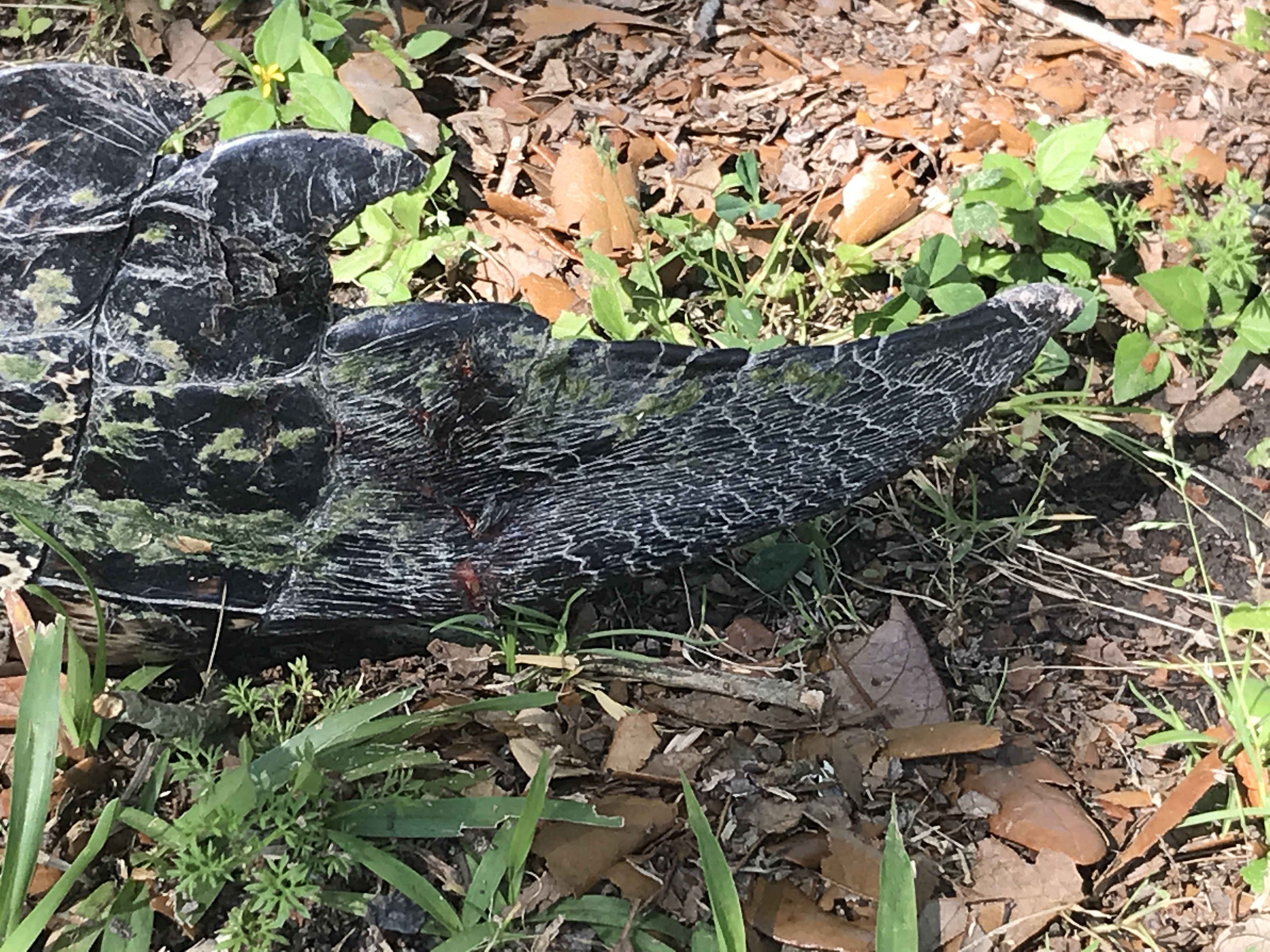

Supplement: Supplementary file 5 — Supplementary Data 1. [file 41598_2020_77052_MOESM5_ESM.zip › SData1/A02_M_RT/A02_M_image_4.jpeg]

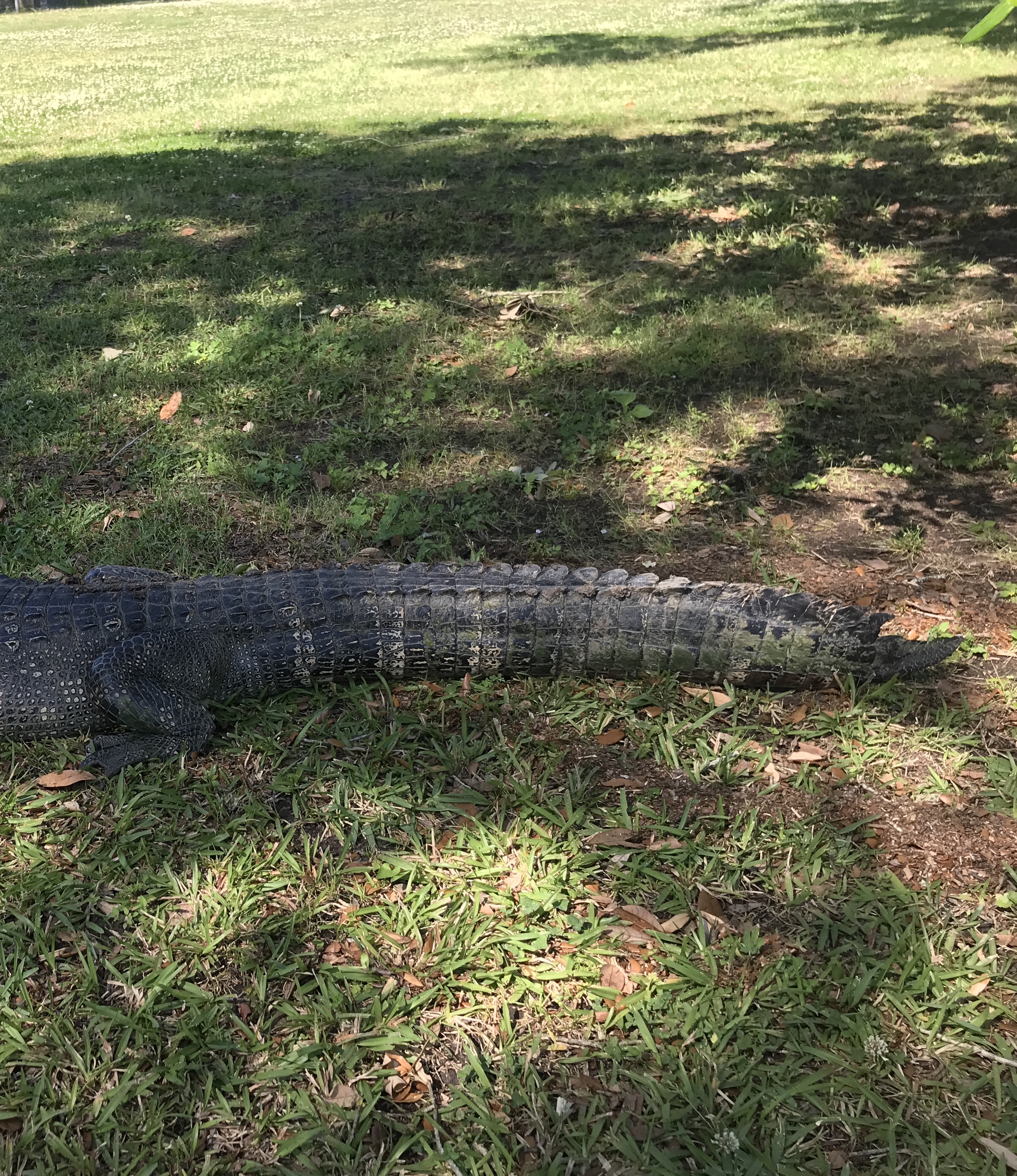

Supplement: Supplementary file 5 — Supplementary Data 1. [file 41598_2020_77052_MOESM5_ESM.zip › SData1/A02_M_RT/A02_M_image_3.jpeg]

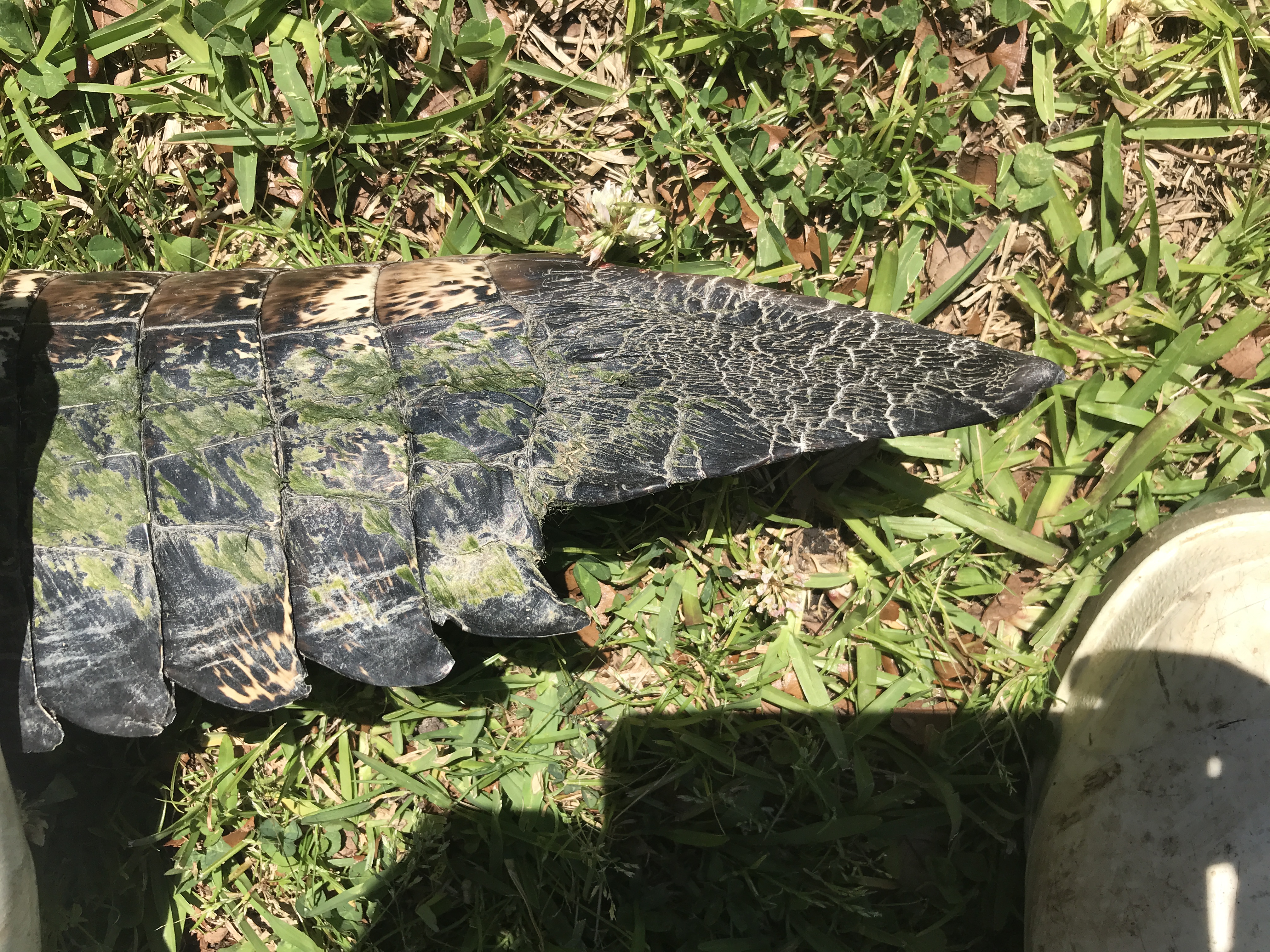

Supplement: Supplementary file 5 — Supplementary Data 1. [file 41598_2020_77052_MOESM5_ESM.zip › SData1/A02_M_RT/A02_M_image_2.jpeg]

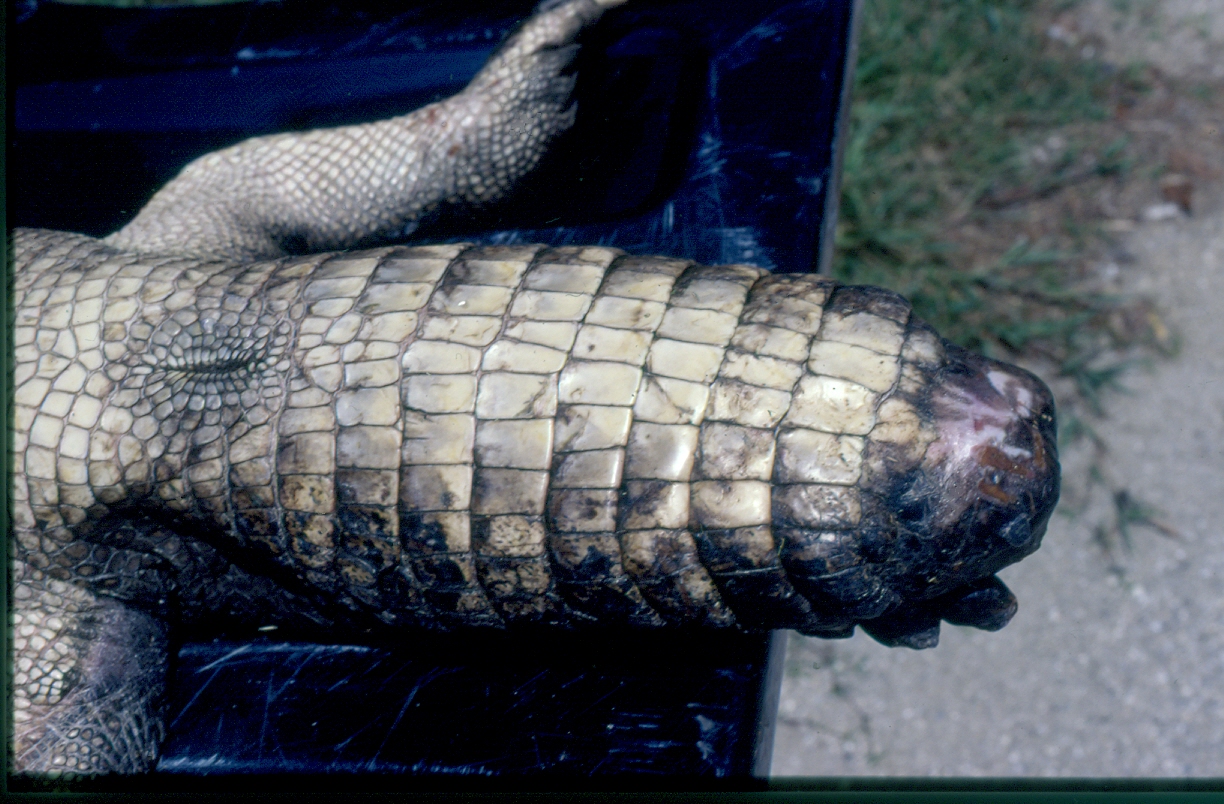

Supplement: Supplementary file 6 — Supplementary Data 2. [file 41598_2020_77052_MOESM6_ESM.zip › SData2/repair_image_8.jpg]

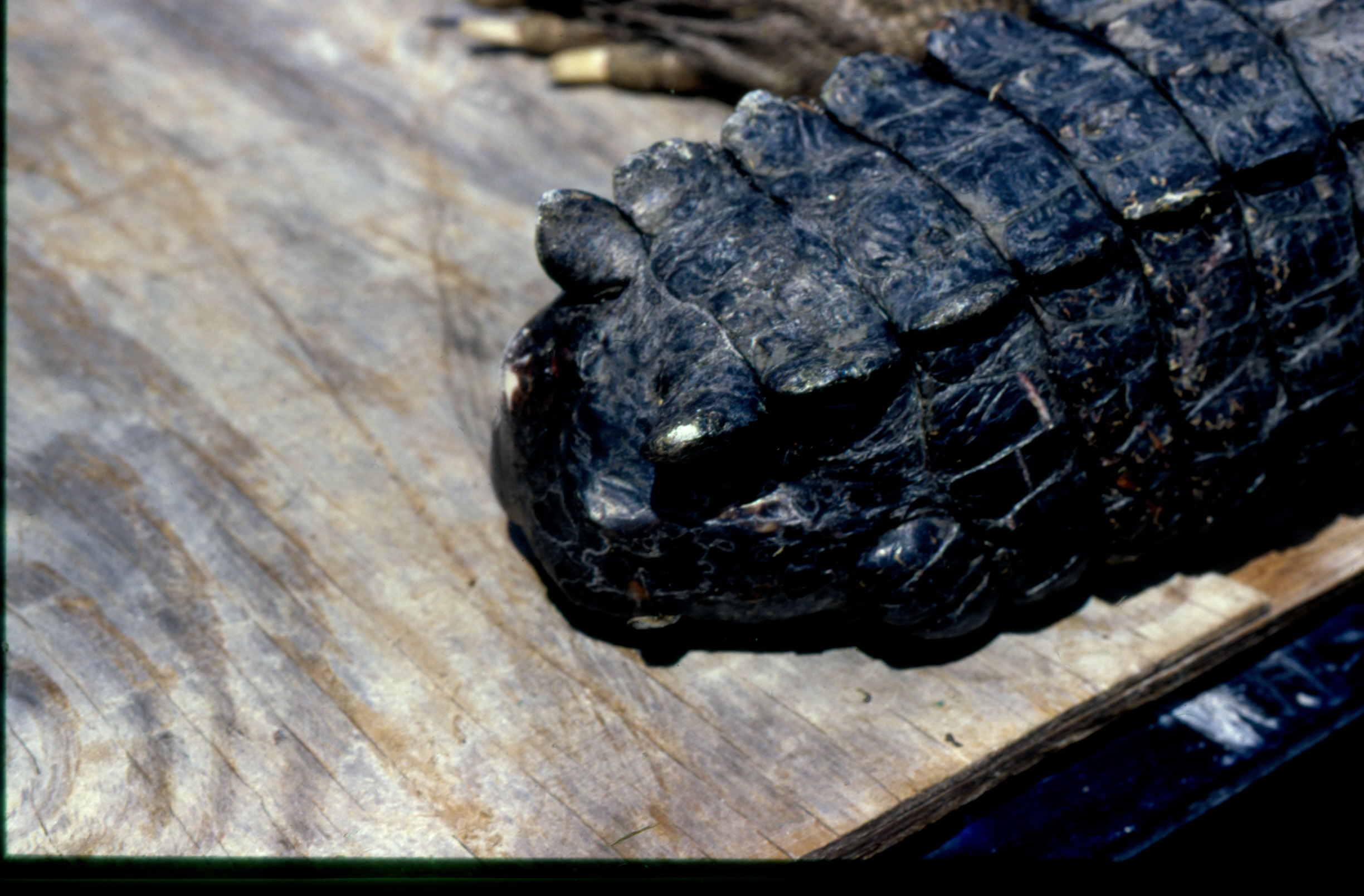

Supplement: Supplementary file 6 — Supplementary Data 2. [file 41598_2020_77052_MOESM6_ESM.zip › SData2/repair_image_6.jpg]

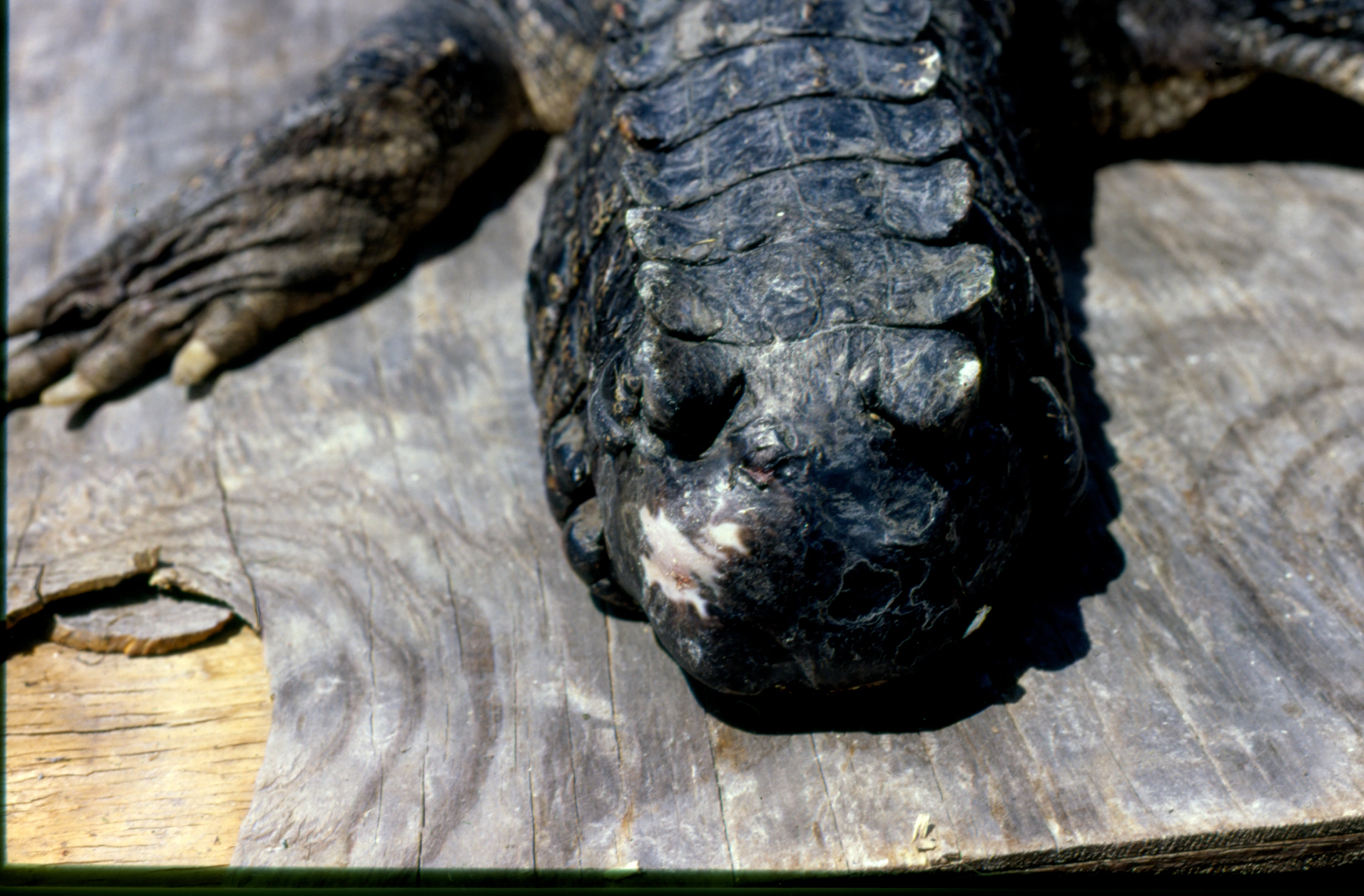

Supplement: Supplementary file 6 — Supplementary Data 2. [file 41598_2020_77052_MOESM6_ESM.zip › SData2/repair_image_7.jpg]

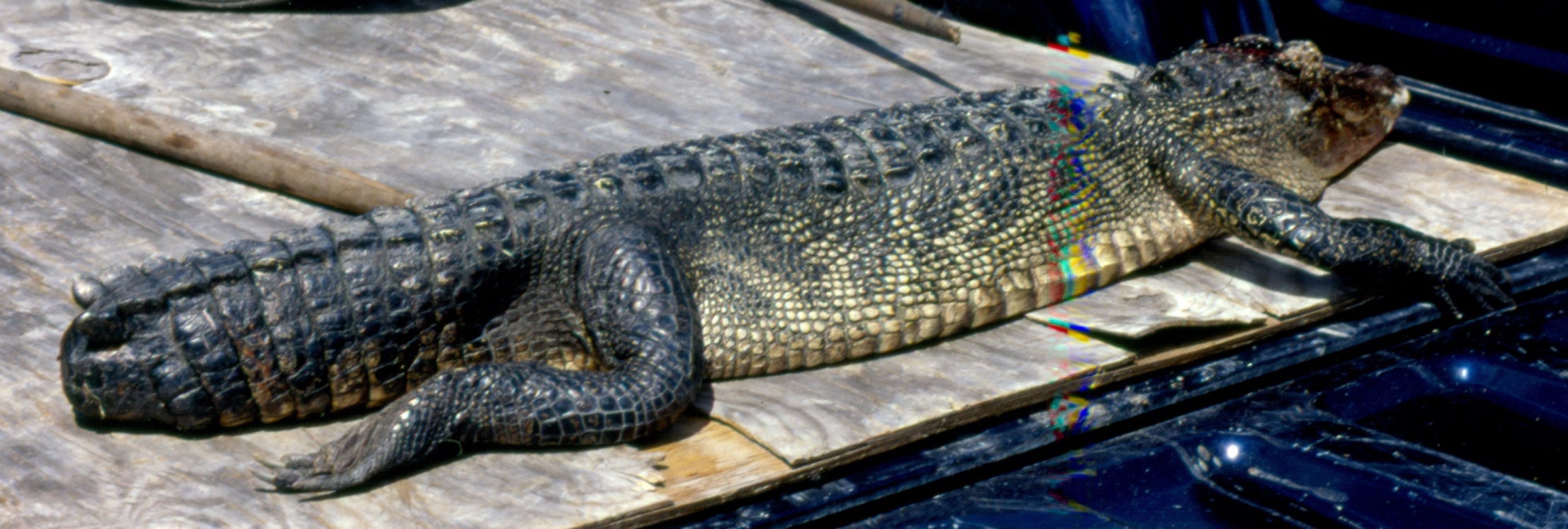

Supplement: Supplementary file 6 — Supplementary Data 2. [file 41598_2020_77052_MOESM6_ESM.zip › SData2/repair_image_5.jpg]

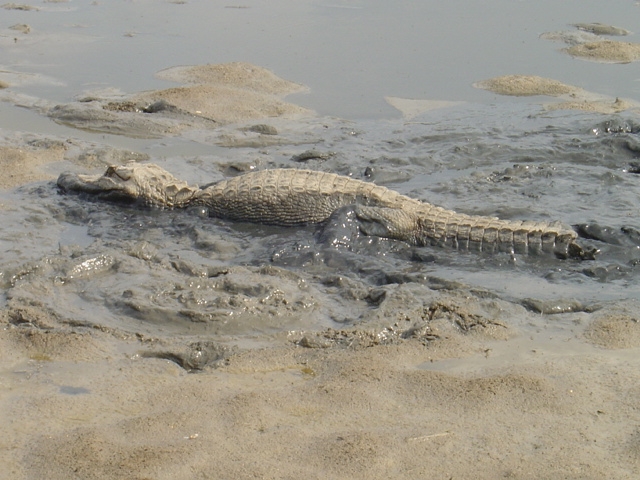

Supplement: Supplementary file 6 — Supplementary Data 2. [file 41598_2020_77052_MOESM6_ESM.zip › SData2/repair_image_4.JPG]

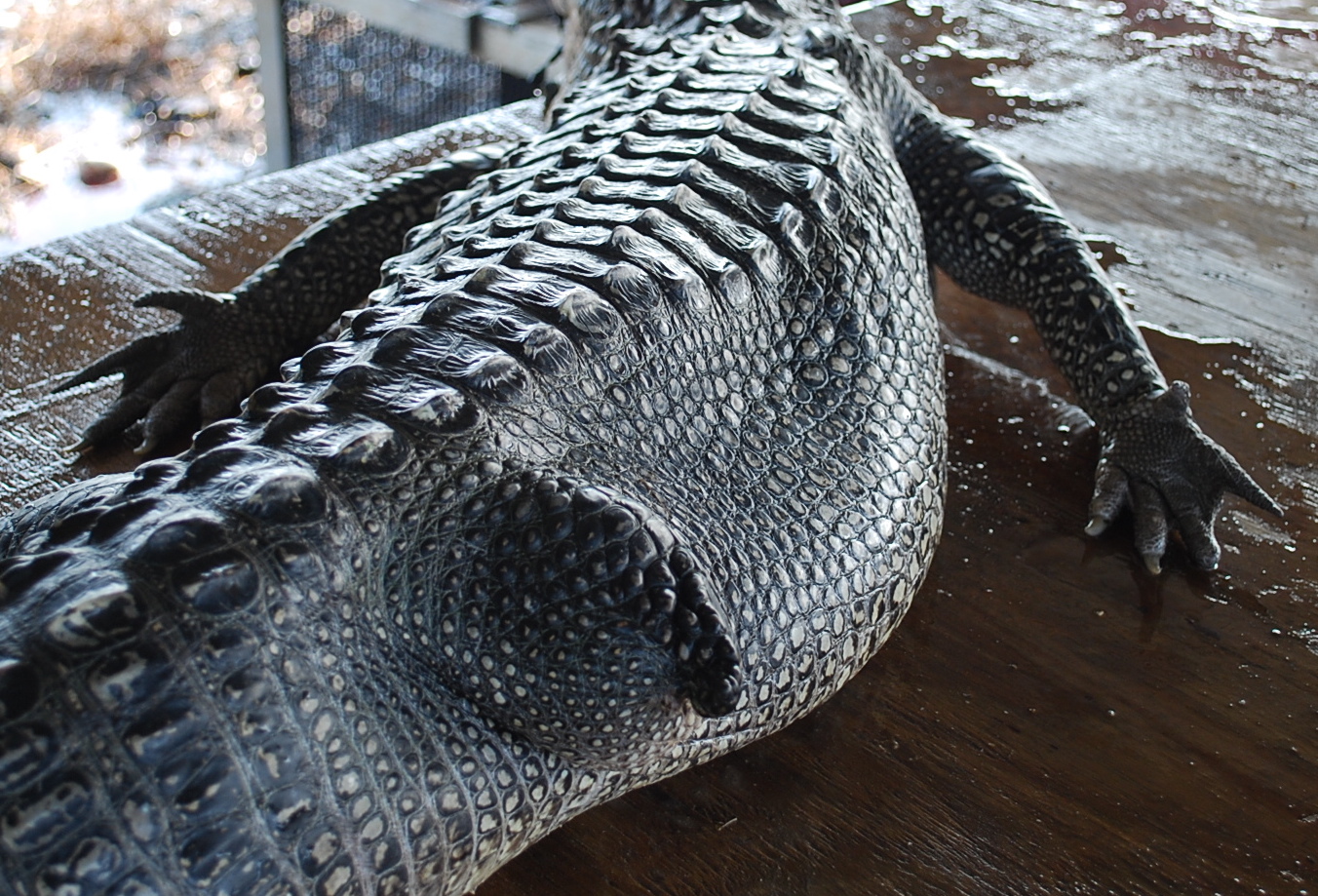

Supplement: Supplementary file 6 — Supplementary Data 2. [file 41598_2020_77052_MOESM6_ESM.zip › SData2/repair_image_1.JPG]

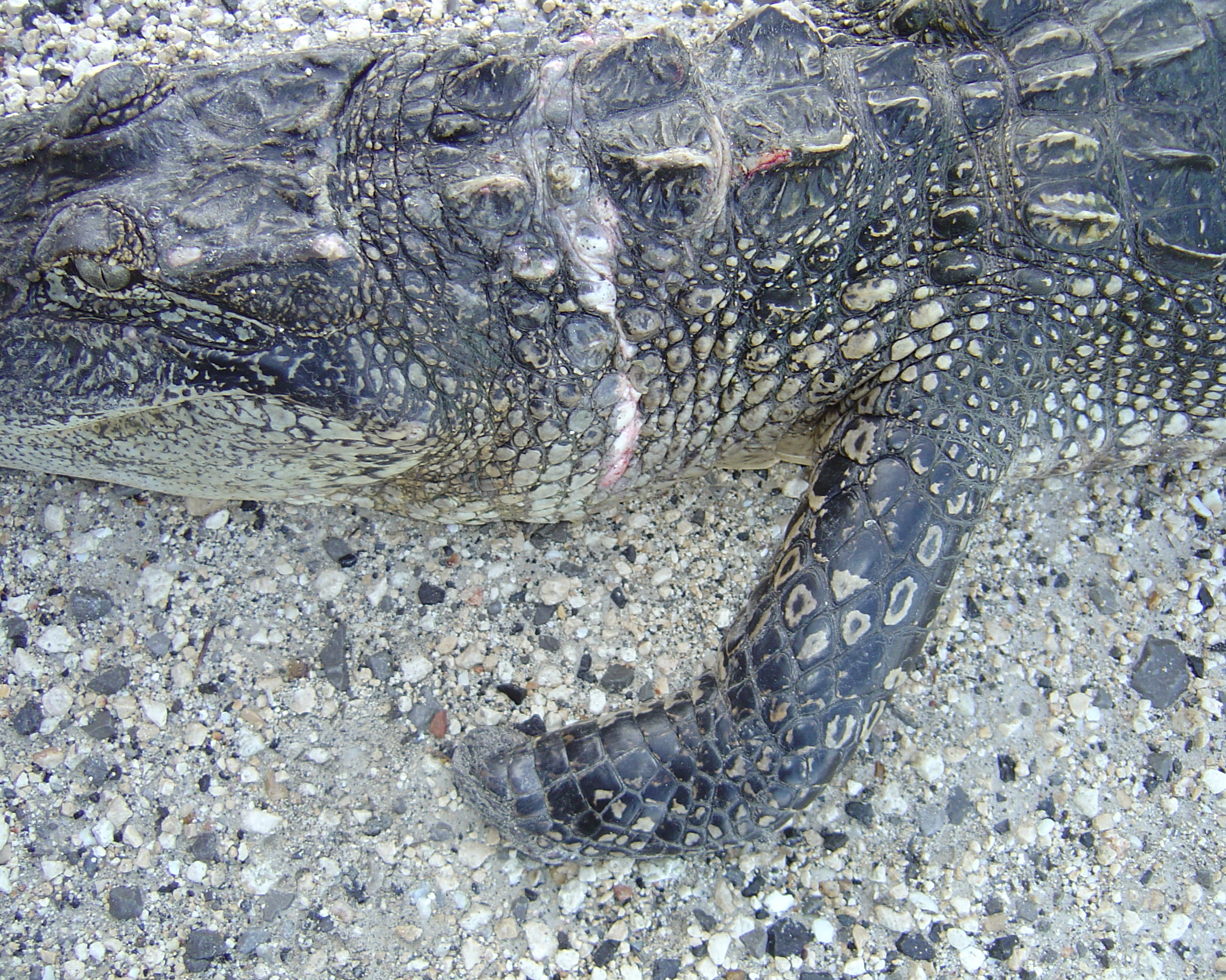

Supplement: Supplementary file 6 — Supplementary Data 2. [file 41598_2020_77052_MOESM6_ESM.zip › SData2/repair_image_3.JPG]

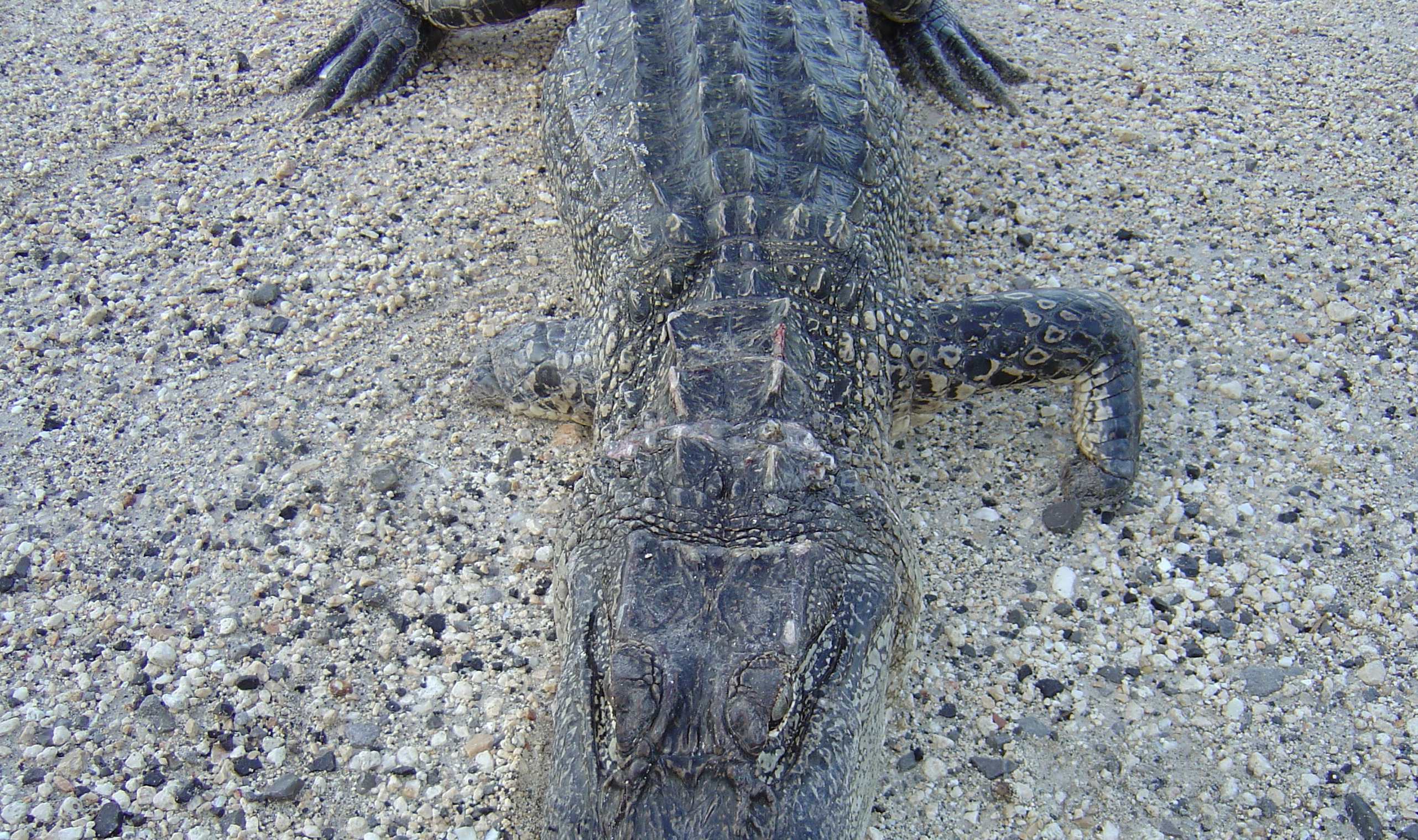

Supplement: Supplementary file 6 — Supplementary Data 2. [file 41598_2020_77052_MOESM6_ESM.zip › SData2/repair_image_2.JPG]
