# Supplementary material for: Anatomical and histological analyses reveal that tail repair is coupled with regrowth in wild-caught, juvenile American alligators (Alligator mississippiensis)
Source: Sci Rep. 2020 Nov 18;10:20122. doi: 10.1038/s41598-020-77052-8 (PMC7674433; doi:10.1038/s41598-020-77052-8)
Supplement: Supplementary file 7 — Supplementary Data 3. [file 41598_2020_77052_MOESM7_ESM.zip › SData3/A04_alligator_male_biopsy_results.pdf]

PATHOLOGY REPORT

BIOPSY

Department of Veterinary Pathology
Louisiana State University, Baton Rouge

PATHOLOGY NO: 04B5556

Grad Asst: Dr. David

Pathologist: Dr. Lomax

Clinician: Dr. Nevarez **Addr:** LSU SVM, Baton Rouge LA 70803 [redacted]

Clin. Ph: 225-578-9600

Owner: Wildlife & Fisheries **Addr:** Furand Refuge Division, New Iberia LA 70560

Own. Ph: 337-373-0032

Species: Exotic **Brd/Str:** **Sex:** **Age:** - **Wt:** -

Animal ID: Alligators 77283 Reptiles ○ D ○ E ◉ B **Date D/E/B:** ~~4~~ 10/21/2004

DISEASE DIAGNOSIS:
No specific disease process observed

biopsy done 10/21/04

MORPHOLOGICAL DIAGNOSIS:
Skin: Abundant dermal collagen

CLINICAL SUMMARY (as provided by clinician):
Tissue Submitted: Biopsy from alligator tail - 3 punch biopsies
Patient history on file
PDD: Tissue Fibrosis

GROSS FINDINGS: ~~4~~ 10/21/2004

MICROSCOPIC FINDINGS:
Three punch biopsies, identified per submitter as originating from the tail of an alligator, are examined. Sections are composed of cartilage, epidermis and dermis. The dermal collagen in all sections is dense, abundant and normally arranged.

LABORATORY RESULTS:

COMMENTS:
(*) Dermal collagen was abundant but appeared normally arranged. If dermal collagen is excessive this could be compatible with the diagnosis of tissue fibrosis; however, the diagnosis cannot be definitively confirmed or ruled out without age, species and location matched controls.

LGL/AD:lw

Resident: [signature] DVM Date: 10/26/04

Pathologist: [signature] DVM Date: 10/26/04

Shark-tail alligator

Sharktail ally

1 of 1
